# Enhancing Access to Mental Health Services for Antepartum and Postpartum Women Through Telemental Health Services at Wellbeing Centers in Selected Health Facilities in Bangladesh: Implementation Research

**DOI:** 10.2196/65912

**Published:** 2025-01-03

**Authors:** Aniqa Tasnim Hossain, Md Hafizur Rahman, Ridwana Maher Manna, Ema Akter, SM Hasibul Islam, Md Alamgir Hossain, Tasnu Ara, Nasimul Ghani Usmani, Pradip Chandra, Maruf Ahmed Khan, SM Mustafizur Rahman, Helal Uddin Ahmed, Muhammad Kamruzzaman Mozumder, Jesmin Mahmuda Juthi, Fatema Shahrin, Sadia Afrose Shams, Fahmida Afroze, Mukta Jahan Banu, Shafiqul Ameen, Sabrina Jabeen, Anisuddin Ahmed, Mohammad Robed Amin, Shams El Arifeen, Mohammad Sohel Shomik, Ahmed Ehsanur Rahman

**Affiliations:** 1 International Centre for Diarrhoeal Disease Research, Bangladesh Dhaka Bangladesh; 2 Director General of Health Services Ministry of Health and Family Welfare Dhaka Bangladesh; 3 National Institute of Mental Health and Hospital Ministry of Health and Family Welfare Dhaka Bangladesh; 4 Department of Clinical Psychology University of Dhaka Dhaka Bangladesh

**Keywords:** Wellbeing Centers, antepartum, postpartum, depression, anxiety, implementation

## Abstract

**Background:**

Globally, 10% of pregnant women and 13% of postpartum women experience mental disorders. In Bangladesh, nearly 50% of mothers face common mental disorders, but mental health services and trained professionals to serve their needs are scarce. To address this, the government of Bangladesh’s Non-Communicable Disease Control program initiated “Wellbeing Centers,” telemental health services in selected public hospitals.

**Objective:**

This study examines implementation outcomes, including adoption, accessibility, acceptability, feasibility, usefulness, need, experience, perception, and expectations of the Wellbeing Centers, with a focus on antepartum and postpartum women.

**Methods:**

Between January 2023 and August 2024, we interviewed 911 antepartum and postpartum women receiving mental health services and 168 health care providers at 6 Wellbeing Centers in 4 districts in Bangladesh. Data collection involved both quantitative and qualitative methods. Implementation outcomes were measured following the World Health Organization’s implementation research framework. Depression and anxiety symptoms were assessed using the Patient Health Questionnaire-9 and Generalized Anxiety Disorder-7 questionnaires. Descriptive statistics and adjusted odds ratios (aORs) with 95% CIs were used to evaluate the implementation outcomes. Qualitative information was obtained through in-depth interviews and key-informant interviews.

**Results:**

Almost all health care providers (165/168, 98.2%) reported that the Wellbeing Centers were feasible to implement in their health facilities; however, about half (84/168, 50%) felt that trained staff to operate them were insufficient. Almost all women agreed that the Wellbeing Centers were acceptable (906/911, 99.8%), useful (909/911, 99.8%), and enhanced access to mental health care (906/911, 99.5%). Patients visiting district-level hospitals had higher odds of access (aOR 1.5, 95% CI 1.1-2.0) to Wellbeing Centers. Moreover, 77.4% (705/911) of women experienced depression symptoms, and 76.7% (699/911) experienced anxiety symptoms. About 51.8% (472/911) experienced tiredness or lack of energy, 50.9% (464/911) felt nervous, anxious, or on edge, 57.2% (521/911) felt worried, and 3.8% (35/911) had suicidal ideation almost every day. Patients visiting district hospitals had higher odds (aOR 2.6, 95% CI 1.8-3.78) of depression and anxiety symptoms compared to the patients visiting subdistrict-level hospitals. Decreasing trends in Patient Health Questionnaire-9 scores (from mean 14.4, SD 0.47 to mean 12.9, SD 0.47) and Generalized Anxiety Disorder-7 scores (from mean 13.3, SD 0.49 to mean 12.5, SD 0.48) between 2 counseling sessions indicated improved mental health in the antepartum and postpartum women. The Wellbeing Centers’ services were appreciated for their privacy and being free and accessible. However, stigma, postpartum illness, and long waiting times prevented some women from using these services.

**Conclusions:**

To our knowledge, this is the first implementation research assessing telemental health in public health facilities involving trained psychologists and psychiatrists. Our study highlighted the increased accessibility, feasibility, acceptability, and utility of Wellbeing Centers for antepartum and postpartum women in Bangladesh, supporting their scale-up in similar settings.

## Introduction

### Background

Maternal mental health problems are common during pregnancy and after birth [1]. It is recognized as a global public health issue, as approximately 10% of antepartum and 13% of postpartum women experiencing some sort of mental health disorders [2]. The prevalence of maternal common mental disorders is high (49%) in Bangladesh, which underscores a need to screen for depression and anxiety symptoms during pregnancy and postpartum period [3,4]. Around 1 in 5 women experience depressive symptoms during pregnancy, and around 1 in 3 women experience anxiety in rural Bangladesh [4]. In a different study, postpartum women in rural Bangladesh reported that 11% had depressed symptoms, 35% had anxiety symptoms, and 3.4% had both depression and anxiety symptoms [5]. A recent study suggested that postpartum depression symptoms have been more common among impoverished rural mothers during the shutdown in Bangladesh [6-8].

### Adverse Effects of Maternal Mental Disorders During the Antepartum and Postpartum Periods

Pregnant women with low education, history of economic difficulties, poor marital relationships, family history of any common mental disorder, poor social and partner support, bad obstetric history, current or previous exposure to violence, preference to have a male child, history of abortions, and disturbed family environment are more likely to report any kind of antepartum and postpartum mental disorders [4,9-13]. Maternal depression may cause negative health-related behaviors and adverse outcomes, including psychological and developmental disturbances in infants, children, and adolescents [14]. Women with severe mental disorders also have increased risks of pre-eclampsia, antepartum and postpartum hemorrhage, placental abruption, impaired intrauterine growth, abortion, and cesarean section, and stillbirths are associated with antepartum and postpartum depression and anxiety [14-19]. Severe mental disorders result in suicide, a leading cause of maternal death in pregnancy and the postpartum period, which contributes to maternal mortality and low quality of life [1,20,21].

### Why Videoconference-Based Counseling Is Appropriate as an Intervention for Maternal Mental Disorders in the Context of Bangladesh

Early detection and treatments are necessary to address these maternal mental health issues. Maternal mental disorders are treatable using effective counseling and therapies [22]. However, the availability and access to mental health services are somewhat limited in rural Bangladesh. In addition, the number of available psychologists and psychiatrists is very low. Bangladesh has an estimated 260 psychiatrists, or approximately 0.16/100,000 population, as well as 700 nurses who provide mental health specialty care (0.4/100,000), and 565 psychologists (0.34/100,000) mostly concentrated in urban settings [23]. Providing in-person mental health care with limited capacities such as very low designated government facilities with few specialty service providers is difficult [23].

However, Bangladesh has very good network coverage, which can be used for telehealth counseling services. Telemental health services, which gained popularity during the COVID-19 pandemic, are also commonly used in Bangladesh [24]. Several studies have found that telephone-based treatment significantly improved short-term symptoms and considerably alleviated the advancement of postnatal depression [25,26]. Evidence suggests that digital psychological interventions for mental health problems in developing countries are effective when usual care for mental health problems is minimal [27]. Another study reported that mothers experienced less maternal depression after receiving videoconference-based counseling [28]. Videoconference-based counseling has emerged as a practical and efficient means of providing mental health treatment in resource-limited communities for reducing symptoms of psychiatric disorders and helping to improve quality of life [29-33].

### Implementation of Wellbeing Centers in Collaboration With the Non-Communicable Disease Control Program of the Government of Bangladesh

Cognizant of this reality, the Non-Communicable Disease Control (NCDC) program of the government of Bangladesh (GoB) initiated the telemental health service called “Wellbeing Centers” in 6 public hospitals of Bangladesh to provide telemental health services with facilitation support from the International Centre for Diarrhoeal Disease Research, Bangladesh (icddr,b). General patients along with women with maternal mental health disorders can take personalized and specialized counseling support from a pool of psychologists and psychiatrists through videoconference counseling at the Wellbeing Centers [34,35]. It is important to know whether these services are adequately benefitting the targeted population in a larger number of facilities for scaling up these Wellbeing Centers in other districts in Bangladesh since no study ever explored it in Bangladesh.

### Aims

The primary aim of this study is to assess the implementation outcomes (feasibility, accessibility, adoption, acceptability, usefulness, need, experience, perception, and expectation) of the Wellbeing Centers in selected district and subdistrict hospitals of Bangladesh. We will also explore the prevalence of depression and anxiety symptoms in the targeted population as a secondary outcome for demonstrating the need for such mental health care.

## Methods

### Study Setting

**Figure 1:**
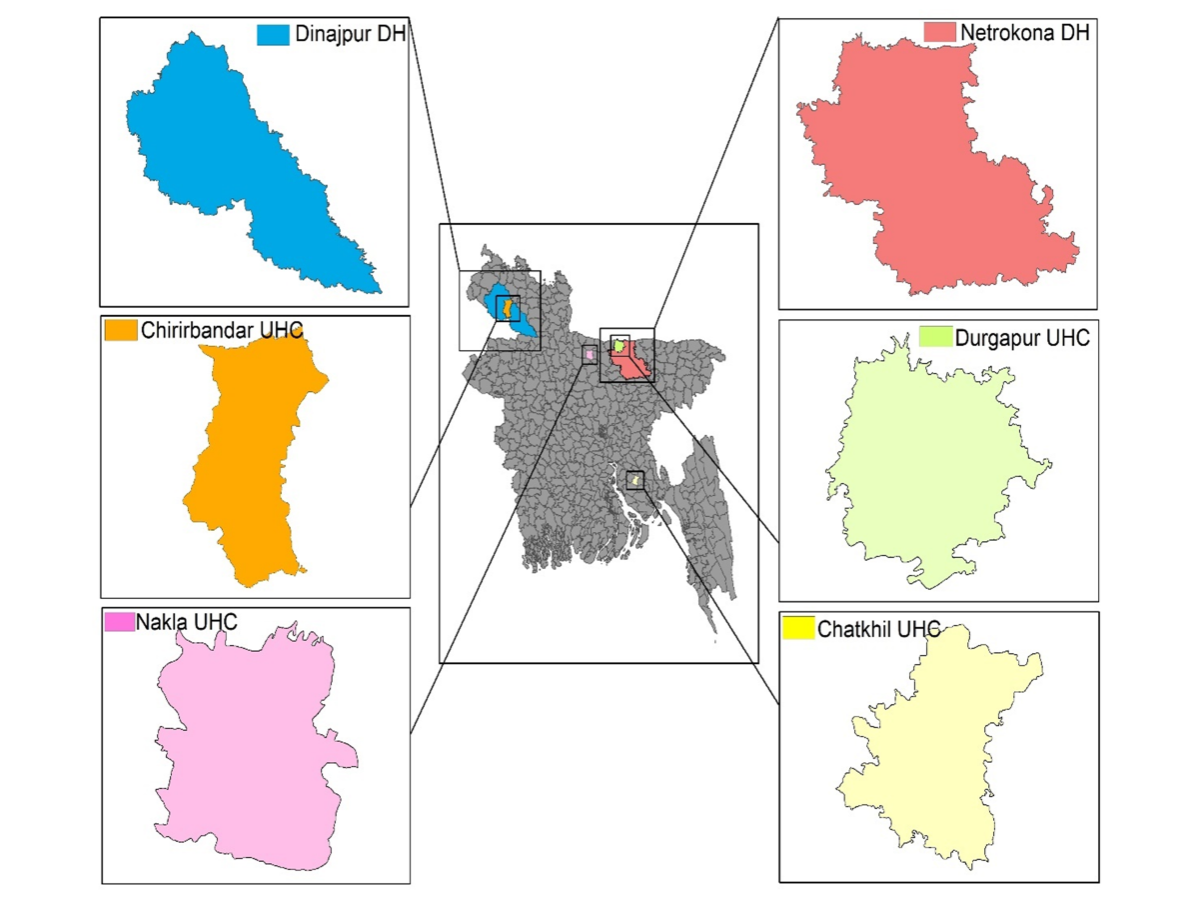
Study sites. DH: district hospital; UHC: upazila health complex.

A total of 6 Wellbeing Centers were implemented in district hospitals (DHs) and 4 *upazila* (subdistrict) health complexes (UHCs) in Dinajpur district of Rangpur division and Netrokona district of Mymensingh division. Two other subdistrict-level health care facilities were from Nakla in the Sherpur district and from Chatkhil in the Noakhali district. Selected health care facilities were Dinajpur DH, Netrokona DH, Durgapur UHC, Chirirbandar UHC, Nakla UHC, and Chatkhil UHC (Figure 1). The NCDC program of the Directorate General of Health Services suggested carrying out the Wellbeing Center services in these enlisted 6 health care facilities.

### Study Design

An implementation research study was conducted, where the NCDC program designed, developed, and demonstrated an implementation model to introduce Wellbeing Centers for providing telemental health services. The study used both quantitative and qualitative data collection. Implementation facilitation support was provided, and assessments were conducted by icddr,b, an international health research organization based in Bangladesh.

### Study Participants

Antenatal and postnatal women who visited to the outpatient settings (mainly antenatal care [ANC] and postnatal care [PNC] corner) and received counseling from Wellbeing Centers of the 6 selected health care facilities were enrolled in this study. A total of 911 women in antepartum and postpartum periods received care from the Wellbeing Centers, and 168 health care facility managers and providers directly involved in the implementation were surveyed and reincluded in this analysis. The health care facility managers and providers included civil surgeons of the corresponding districts, hospital superintendents of the corresponding DHs, subdistrict health and family planning officers of the corresponding UHCs, resident medical officers (RMOs) of corresponding DHs and UHCs, physicians, and gynecological consultants from the outpatient departments.

### Development of the Wellbeing Centers at the Health Care Facilities

#### Overview

The NCDC program of the Directorate General of Health Services, Ministry of Health and Family Welfare of Bangladesh received implementation support from the icddr,b along with other institutions, such as the National Institute of Mental Health (NIMH) and Department of Clinical Psychology, University of Dhaka, to establish the Wellbeing Centers.

#### Creating a Pool of Psychologists and Psychiatrists

A pool of trained psychologists and psychiatrists has been formed to deliver mental health care services. This pool of psychologists and psychiatrists was guided and mentored by professional bodies from clinical and counseling psychology and psychiatry.

#### Establishment of the Wellbeing Centers

Equipment and technology recourses included a computer, an internet connection, and a webcam in each Wellbeing Center at the facility. To establish an internet connection, an internet router was provided. In cases of electrifying fall, an uninterrupted power supply was used. Psychologists and psychiatrists and patients were provided with headphones to cancel or isolate ambient noise. With the help of a digital platform, appointments were scheduled. Patients were connected with the psychologists through videoconferencing. In the hospital, a room was allocated for the Wellbeing Center. This room was dedicatedly used for telemental health, maintaining appropriate privacy and confidentiality. The webcam and video monitor were placed at the client’s eye level to best approximate a face-to-face interaction.

#### Training

Health care administrators, expertise in mental health, along with facility managers partnered with icddr,b to facilitate training and workshops for district and subdistrict-level health care providers (such as RMOs, physicians, gynecological consultants, and health workers). Additionally, the implementation support team organized the orientation of both government and program-supported health workers to promote the Wellbeing Center activities. Training content covered the use of depression and anxiety screening tools, patient reception, appointment scheduling, communicable liaison, the integration of mental health services in outpatient settings, effective web-based patient engagement, the use of technology in delivering mental health services, patient referral processes, follow-ups, and crisis management.

#### Service Provision

At first, women who came to seek health care service at ANC or PNC corners of the health care facilities were referred by the physicians or gynecological consultants to the Wellbeing Centers. Afterward, a health worker screened and redirected women of antepartum and postpartum to a Wellbeing Center and registered the client’s through the digital platform using their name and phone number. The health worker noted the availability of psychologists, and an appointment was then fixed. By creating a digital meeting link, the patient’s information and schedule were shared with the psychologists. Patients were supported by the health worker to prepare and make necessary arrangements for connecting to the psychologists through videoconference ensuring adequate privacy.

**Figure 2:**
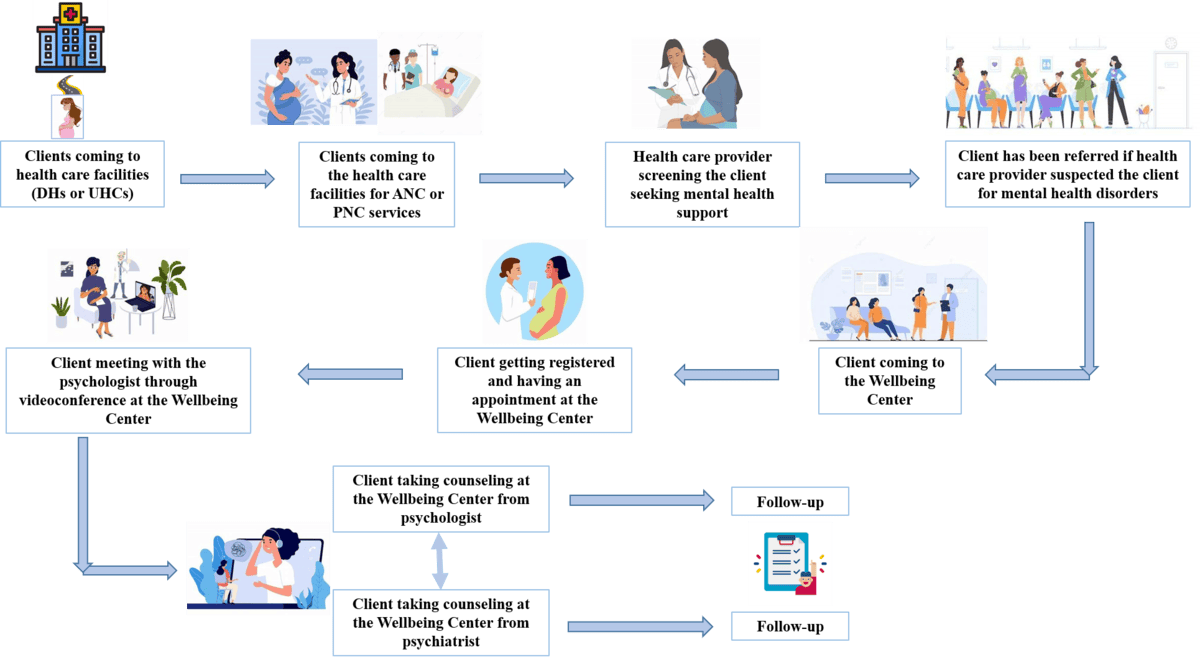
Mental health service delivery mechanism of Wellbeing Centers. ANC: antenatal care; DH: district hospital; PNC: postnatal care; UHC: upazila health complex.

Psychologists assessed clients’ mental health disorders using psychometric tools and then provided tailored counseling. Psychologists designed additional management plans and follow-ups based on the clients’ improvement dimensions. When counseling proved insufficient to address moderate to severe instances, clients were referred to the NIMH’s psychiatrists. Then, the health worker made an appointment and used videoconference-based counseling to connect with the psychiatrist for further treatment. Each client had a second screening by a health worker using the Patient Health Questionnaire-9 (PHQ-9) and Generalized Anxiety Disorder-7 (GAD-7) during follow-up sessions in order to measure the degree of change in their mental condition (Figure 2).

### Data Collection

Both quantitative and qualitative methods were undertaken to collect data. Quantitative data were collected using tablets, and qualitative interviews were done using audio recorders. We developed a data entry interface for this implementation research to manage data. A quantitative survey was conducted among women by trained health workers. A structured quantitative questionnaire was used to collect data on demographics [36,37], validate depression and anxiety symptom screening in outpatient settings [38-41], and assess implementation outcomes including acceptability, usefulness, and adoptability [42]. Assessment of feasibility among health facility managers was determined using the World Health Organization’s (WHO) improving health system and services for mental health guideline [42]. Qualitative information was received by trained researcher using in-depth interviews (IDIs) and key informant interviews (KIIs). All data collection tools are presented in detail in Multimedia Appendices 1-4. About 10 qualitative IDIs were conducted on experiences, perceptions, and expectations regarding the videoconference-based mental health counseling among women who received ANC and PNC in the health care facilities at the Wellbeing Centers. Moreover, 15 KIIs were conducted among the health care facility managers, RMOs, physicians, psychologists, psychiatrists, and health workers. The number of antepartum and postpartum women who took follow-up sessions was 51.

### Study Measures

Basic demographic information included age (years), types of care (ANC and PNC), religion, profession, education (years completed), household income (taka per month), and catchment area (subdistrict and district) were determined. The WHO’s implementation research in health care guideline was followed in terms of defining acceptability, usefulness, feasibility, and adoption [42]. Table 1 provides detailed indicator information for all the implementation outcomes assessed in this study.

**Figure 3:**
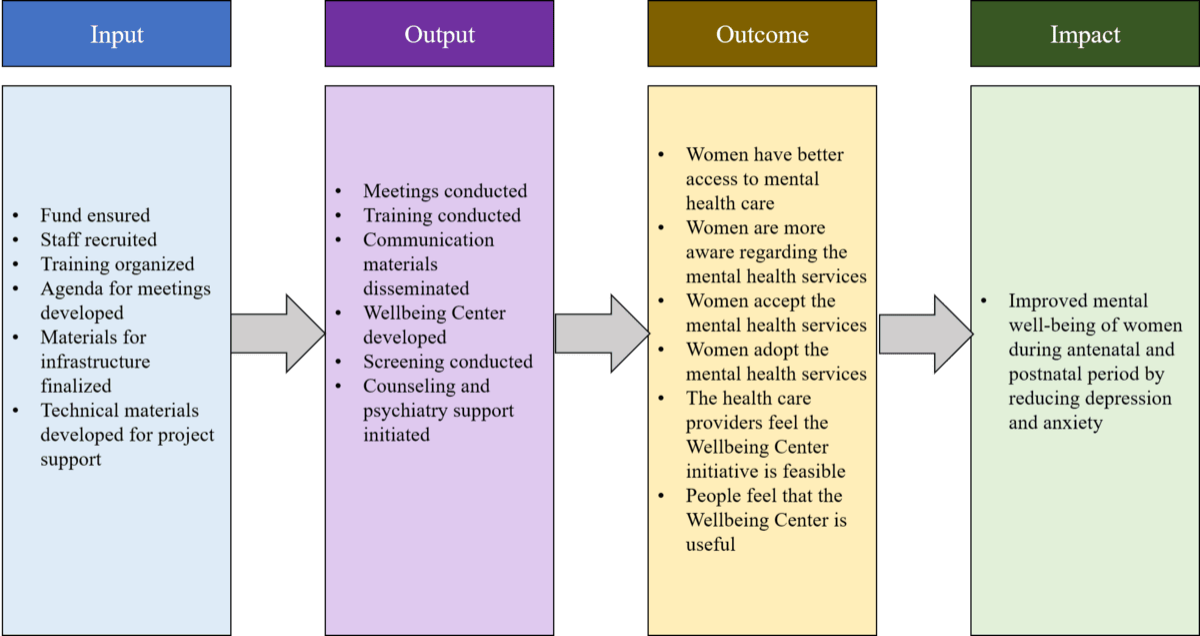
Logical framework of the telemental health intervention.

The PHQ-9 [43] and the GAD-7 scales [44] were used to evaluate depression and anxiety in outpatient settings, respectively [39,41]. The PHQ-9 is a 9-item questionnaire that assesses depression symptoms in a range of 0=not at all to 3=nearly every day. The PHQ-9 score ranges from 0 to 27, with mild, moderate, moderately severe, and severe depression symptoms equating to cutoff values of 5, 10, 15, and 20, respectively. The GAD-7 is a 7-item questionnaire that measures anxiety symptoms on a range of 0=not at all to 3=nearly every day. The GAD-7 scale has a score range of 0 to 21, with mild, moderate, and severe anxiety symptoms equating to cutoff values of 5, 10, and 15, respectively. Figure 3 presents the logical framework of the telemental health intervention in reducing the common mental health disorders among pregnant and postpartum women.

**Table 1:** Indicators according to the objectives for all the implementation outcomes assessed in the study.

Number	Objectives	Study method	Implementation outcome	Indicators or themes
1	To assess the feasibility of the Wellbeing Center at the district-level facility	Quantitative	Feasibility	Percentage of facility managers who feel that Wellbeing Center is implementable in the facility Percentage of facility managers who feel that they have sufficient trained staff in their facility to implement the Wellbeing Center
2	To assess the accessibility to mental health care among antenatal and postnatal women by introducing Wellbeing Center’s telemental health care	Quantitative	Accessibility	Percentage of users who agreed that Wellbeing Center has improved their access to mental health services
3	To assess the adoption of the Wellbeing Center at district-level facility for antepartum and postpartum women	Quantitative	Adoption	Number of women receiving services from the Wellbeing Center
4	To assess the acceptability of the Wellbeing Center at district-level facility for antepartum and postpartum women	Quantitative	Acceptability	Percentage of users who agreed that mental health services from the Wellbeing Center are acceptable to them
5	To assess the usefulness of the Wellbeing Center at district-level facility for antepartum and postpartum women	Quantitative	Usefulness	Percentage of users who agreed that the Wellbeing Center is useful Change in depression and anxiety symptoms scores from first follow-up to second follow-up
6	To assess the proportion of target women with symptoms of depression	Quantitative	Need	Percentage of users who had depressive symptoms
7	To assess the proportion of target women with symptoms of anxiety	Quantitative	Need	Percentage of users who had symptoms of anxiety
8	To assess the experience, perception, and expectation about the telemental health counseling at the district-level facility for the antepartum and postpartum women	Qualitative	Experience, perception, and expectation	Experience, perception, and expectation about the Wellbeing Center

### Data Analysis

#### Quantitative Analysis

Stata (version 15.0; StataCorp) was used for this analysis. We have presented descriptive statistics (frequency and percentage) with 95% CIs. For measuring the effect of various factors (age, types of care, religion, profession, education, household income, and catchment area) on the accessibility of the Wellbeing Centers and need-related indicators (depression, anxiety, and both depression and anxiety), 4 separate fitted models were constructed. Multiple logistic regression models were applied to compute the adjusted odds ratios (aORs) with 95% CI. In the adjusted models, accessibility was considered if a woman “strongly agreed” that the Wellbeing Center increased the accessibility of mental health services. Severe depression symptoms were coded as “1,” and others (mild, moderate, and moderately severe depression) were coded as “0.” For anxiety, severe anxiety symptoms were coded as “1,” and mild and moderate anxiety symptoms were coded as “0.” When a participant was found to have both severe anxiety and depression, we documented this as the co-occurrence of the 2 conditions using a binary response format (1 and 0). Wald statistics were used to assess the model adequacy. We presented the differences in depressive and anxiety symptoms of antepartum and postpartum women occurring nearly every day using radar plots. At the 5% level of significance, the statistical significance of the estimates has been reported.

#### Qualitative Analysis

All audio-recorded interviews, supplemented with field notes, were transcribed verbatim. The transcriptions were then read through several times by all the researchers to get more familiar with the data. The transcriptions were manually thematically analyzed using an inductive approach [45,46]. The three stages of the analysis included (1) reading the interview transcripts; (2) highlighting the related words, coding them in relation to the text, and thereafter classifying them; and finally, (3) identifying the themes with reflective notes. The data were coded and categorized according to the emerging themes. Data were analyzed by NVivo (QSR International) qualitative data analysis software. To respect the anonymity of each participant, no personal identifying information was presented in the result.

### Ethical Considerations

The icddr,b Institutional Review Board granted the study ethics approval (protocol PR-22103). All the eligible women have given written informed consent prior to the enrollment. All data were anonymized or deidentified. No monetary compensation was provided to participants for this research. Consent has been granted from identifiable individual features of research participants or users in any images of the manuscript or supplementary material.

## Results

### Quantitative Findings

The selection process of the patients from the facilities with Wellbeing Center for antepartum and postpartum women is shown in Multimedia Appendix 5. Between January 2023 and August 2024, 16,203 patients visited the outpatient department, from whom 5863 general patients received services from Wellbeing Centers. Among them, 4450 women visiting the ANC and PNC corners received services from 6 Wellbeing Centers. We have considered only the 911 antepartum and postpartum women who received mental health services at the Wellbeing Center.

Table 2 presents the background characteristics of the antepartum and postpartum women who received services from Wellbeing Centers. The majority of the women were young adults aged 20-24 years. Most of the women (n=817, 89.7%) who received mental health services at the Wellbeing Centers and NIMH were referred during ANC visits. Only 2.2% (n=20) of the women were involved in any income-generating activities. In total, 54.6% (n=497) of the women completed secondary-level education, and 46.9% (n=427) were from the low-income group. A total of 70.6% (n=643) of the counseling receiving women visited the subdistrict-level facility.

**Figure 4:**
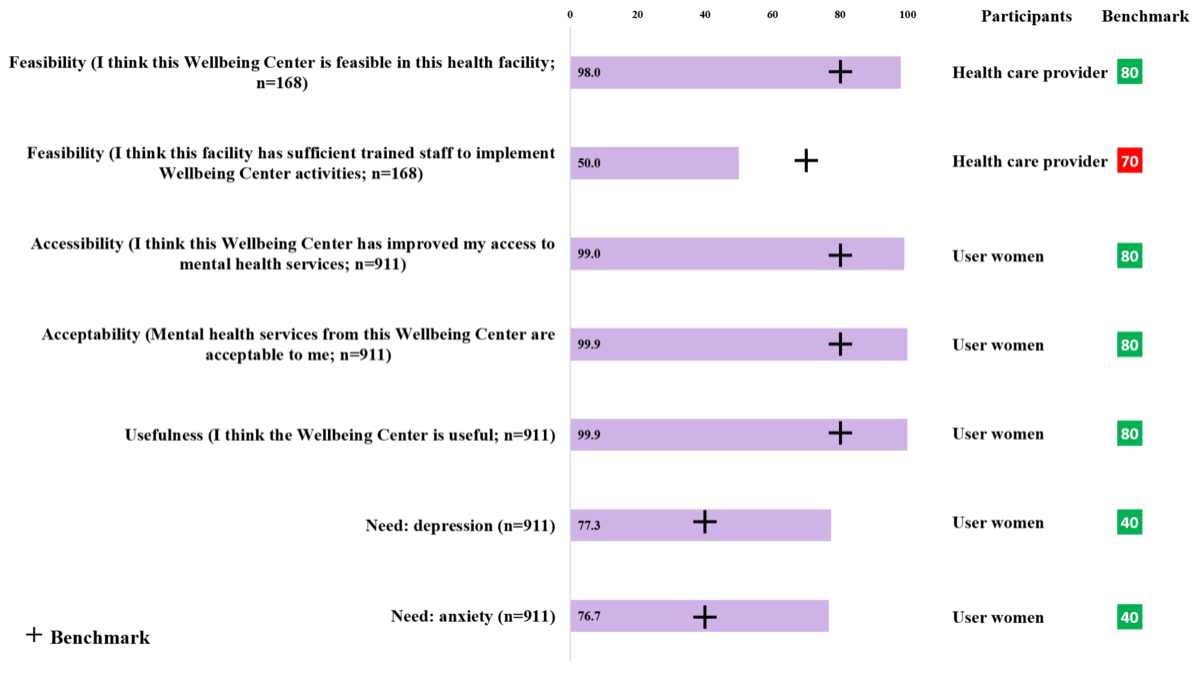
Implementation outcomes of Wellbeing Centers according to the World Health Organization guidelines.

Figure 4 presents the WHO-guided implementation outcomes, feasibility, accessibility, acceptability, usefulness, and need of the Wellbeing Center. Among 168 providers, almost everyone (165/168, 98.2%) reported that the Wellbeing Center is implementable at the facilities. Half of the providers (84/168, 50%) agreed that the facilities have trained staff to maintain the Wellbeing Center. Among the users, almost all antepartum and postpartum women agreed that the Wellbeing Center is increasing accessibility, and it is acceptable and useful for them, as antepartum and postpartum women experience depression and anxiety throughout the period. Around three-fourths of the users had moderate to severe anxiety or depressive symptoms, which demonstrated the need for mental health care.

**Figure 5:**
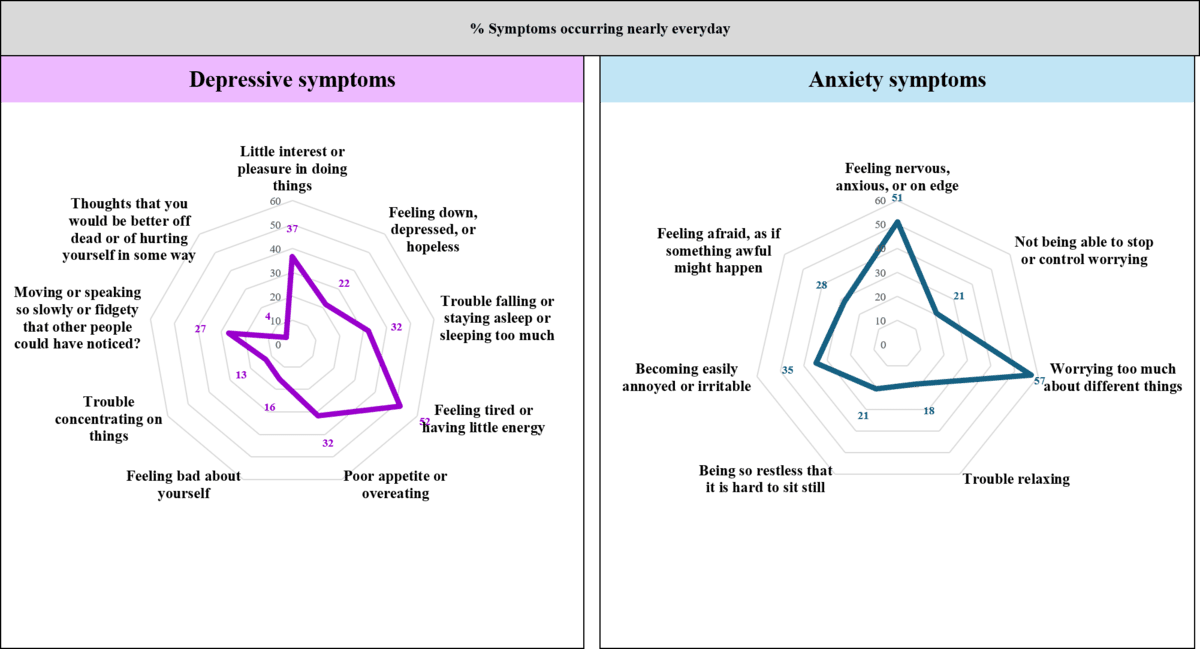
Need for Wellbeing Centers according to the frequency of depressive and anxiety symptoms.

Figure 5 presents the percentage of women who experienced depressive and anxiety symptoms nearly every day in the past 2 weeks, as indicated by the PHQ-9 and GAD-7 scales. Half of the women (472/911, 51.8%) experienced a lack of energy nearly every day, and 36.6% (333/911) experienced a lack of interest and pleasure. Around one-third of women faced issues with trouble falling asleep or sleeping too much and poor appetite or overeating almost every day. In total, 3.8% (35/911) had suicidal ideation almost every day. Among the anxiety symptoms, 57.2% (521/911) worried too much about different issues, and 50.9% (464/911) experienced nervousness almost every day in the past 2 weeks.

The health care providers reported the need for telemental health in their respective facility:

Due to the shortage of well-trained psychiatrists nearby, we have to take treatment from the divisional level health facilities, which is time-consuming and costly. Ensuring proper mental health service district and upazila-level hospital requires service like tele-mental.

KII-15, health worker, Nokla UHC, age 42 years

Table 3 summarizes the effect of various factors on accessibility and need-related indicators. The odds of increased perceived accessibility were lower among patients receiving PNC compared to ANC with aOR 0.45 (95% CI 0.27-0.74). Women with lower education and lower income had higher perceived accessibility. Patients visiting DHs had higher odds of perceived accessibility (aOR 1.48, 95% CI 1.1-2.0). Patients visiting DHs had 3 times higher odds (aOR 2.58, 95% CI 1.82-3.68) of experiencing both depression and anxiety symptoms, expressing the need for mental health services through the Wellbeing Center.

**Figure 6:**
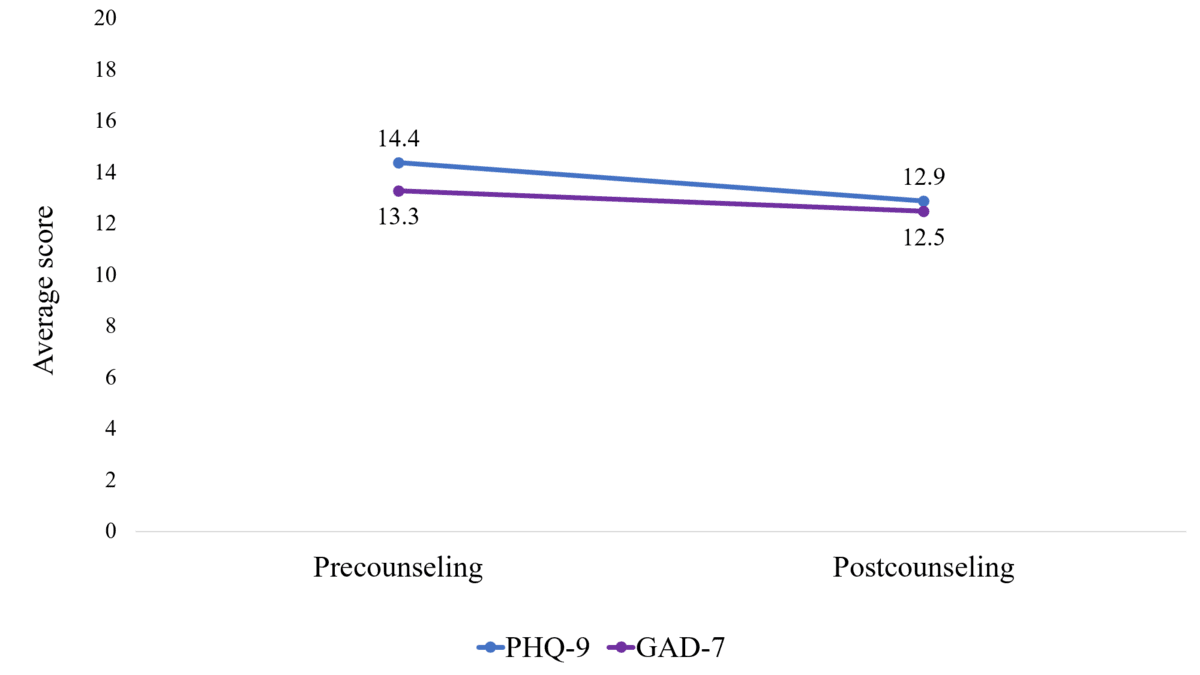
The usefulness of the counseling in reducing the average PHQ-9 and GAD-7 scores (n=51). GAD-7: Generalized Anxiety Disorder-7; PHQ-9: Patient Health Questionnaire-9.

Figure 6 presents the change in average scores of PHQ-9 and GAD-7 between the first and second counseling sessions of the antepartum and postpartum women. The average PHQ-9 score decreased from 14.4 (SD 0.47) to 12.9 (SD 0.47), and the average GAD-7 score decreased from 13.3 (SD 0.49) to 12.5 (SD 0.48) between these 2 sessions, indicating the usefulness of Wellbeing Center. These changes were statistically significant with *P*<.001.

The proportion of people taking follow-up counseling is shown in Multimedia Appendix 6. Only 15.1% (51/338 suggestions of follow-up visits) took follow-up counseling among the patients who were suggested a follow-up session.

**Table 2:** Background characteristics of the antepartum and postpartum women who received services from Wellbeing Centers (N=911).

Background characteristics		Participants, n (%)
**Age (years)**		
	15-19	229 (25.1)
	20-24	332 (36.4)
	25-29	218 (23.9)
	≥30	132 (14.5)
**Type of contact care points at the facility**		
	ANC^a^	817 (89.7)
	PNC^b^	94 (10.3)
**Religion**		
	Muslim	848 (93.1)
	Other^c^	63 (6.9)
**Profession**		
	Housewife	856 (94)
	Involved in income-generation activities	20 (2.2)
	Other^d^	35 (3.8)
**Education (years completed)**		
	No education	12 (1.3)
	Primary	159 (17.5)
	Secondary	497 (54.6)
	Above secondary	243 (26.7)
**Household income**		
	Low	427 (46.9)
	Middle	217 (23.8)
	High	267 (29.3)
**Type of facility location**		
	Subdistrict	643 (70.6)
	District	268 (29.4)

^a^ANC: antenatal care.

^b^PNC: postnatal care.

^c^Hindus, Buddhists, and Christians.

^d^Unemployed and unable to work due to disability.

**Table 3:** Factors associated with accessibility (strongly agree) and need (severe depression and anxiety symptoms) of Wellbeing Center.

				Accessibility					Both depression and anxiety		
				aOR^a^ (95% CI)		*P* value		aOR (95% CI)			*P* value
**Age (years)**											
		15-19	Reference		Reference		Reference			Reference	
		20-24	1.09 (0.76-1.57)		.63		1.42 (0.90-2.23)			.13	
		25-29	0.78 (0.52-1.17)		.22		1.01 (0.60-1.71)			.97	
		≥30	1.14 (0.72-1.81)		.57		0.87 (0.47-1.60)			.65	
**Type of contact care point at the facility**											
		ANC^b^	Reference		Reference		Reference			Reference	
		PNC^c^	0.45 (0.27-0.74)		.002		0.88 (0.48-1.62)			.68	
**Religion**											
		Muslim	Reference		Reference		Reference			Reference	
		Other^d^	0.56 (0.31-1.01)		.05		0.38 (0.15-0.99)			.047	
**Profession**											
	Housewife		Reference		Reference		Reference			Reference	
	Involved in incom- generation activities or other		1.04 (0.57-1.92)		.89		1.06 (0.48-2.32)			.89	
**Education**											
	No education or primary		Reference		Reference		Reference			Reference	
	Secondary		0.40 (0.27-0.58)		.001		1.02 (0.64-1.64)			.93	
	Above secondary		0.44 (0.28-0.69)		.001		0.89 (0.51-1.55)			.67	
**Household income**											
	Low		Reference		Reference		Reference			Reference	
	Middle		0.70 (0.49-0.99)		.047		0.97 (0.62-1.51)			.90	
	High		1.12 (0.81-1.56)		.50		1.07 (0.70-1.62)			.76	
**Type of facility location**											
	Subdistrict		Reference		Reference		Reference			Reference	
	District		1.48 (1.10-2.00)		.010		2.58 (1.82-3.68)			.001	

^a^aOR: adjusted odds ratio.

^b^ANC: antenatal care.

^c^PNC: postnatal care.

^d^Hindus, Buddhists, and Christians.

### Qualitative Findings

#### Perception and Experience Regarding Wellbeing Center Services During the Antepartum and Postpartum Periods

##### Positive Attitude of the Service Providers

The positive attitude of psychologists and health workers of the Wellbeing Centers strengthened the telemental health services compared to other health services. The health workers of the center cordially received the antepartum and postpartum women who were referred by the physicians. During the counseling session, psychologists listened to their problems and issues attentively and provided video counseling, which gives them better feelings about the service at the Wellbeing Center. A pregnant mother mentioned the following:

I feel scared to share my all problems with a physician! I just responded to what the doctor wanted to know. But in the audio-visual call, a counsellor gives a welcoming tone which influenced me to share my mental health issues clearly.

IDI, female, age 32 years

##### Cost and Accessibility

Earlier, antepartum and postpartum women who experienced mental health disorders had to get treatment from the regional medical college hospitals or have to go to specialized hospitals. Because of the Wellbeing Center, people can get mental support on their doorstep. From the Wellbeing Center, anyone can get free-of-cost and hassle-free treatment. Those who seek mental health treatment from private hospitals have to spend considerably more money on paying psychiatrist fees, unethical financial gain of clerks, medication, transportation costs, etc. Nevertheless, patients found videoconference-based counseling at the Wellbeing Centers more convenient than the traditional treatment system, as they do not require any treatment costs, and it provides easy treatment access at their doorsteps. A service receiver mentioned the following:

If I come here [Wellbeing Center] it will save my money and I get treatment from a good specialist that won’t cost me money.

IDI, female, age 22 years

##### Privacy and Confidentiality

While the antepartum and postpartum women received counseling, nobody was present in the room to ensure privacy and confidentiality. Service receivers’ privacy is a prime concern at the Wellbeing Centers, as patients receive services in a separate room that ensures privacy during the counseling. A service receiver said the following:

During in-person consultations, I did not freely communicate to the doctor [psychiatrist-private chamber] because of the other patient’s presence in the waiting area. But in the Wellbeing Center, I do not have worries about violation of [my] privacy while getting audio-visual counseling in a separate room.

IDI, female, age 24 years

##### Name

The name of the mental health service center is Wellbeing Center to overcome the stigma and social taboo associated with psychological support needs. Whenever antepartum and postpartum women and their caregivers come to the corner, they do not hesitate to seek care. This may be due to the perception of a safe and supportive space that encourages open discussion of mental health issues without fear of judgment. The term “well-being” did not demonstrate any stigma. A health worker mentioned the following:

*We do not use the term mental health corner, instead we use “Mon-Shastho Kendro” [Wellbeing Center] to avoid the stigma. When doctors refer them to the Wellbeing Centerthey do not feel hesitation.* [KII, health workers, age 33 years]

#### Barriers to Using the Wellbeing Center Services

##### Stigmas and Taboos

During the pregnancy period, mothers experience numerous negative emotions. Though they can understand that their mental condition is changing, they cannot share their problems due to stigmas and taboos. Even more, they do not disclose their mental health problems to their husbands and family members. Fear of family violence and potential disruption of marriage often prevent pregnant women from disclosing their mental health issues. A pregnant mother mentioned the following:

I do not share my problems with my family members, if I share my mental problems with them then they may make any comments on this that will be very frustrating to me.

IDI, pregnant mother, age 29 years

##### Postpartum Illness

During the postnatal period, mothers are unable to receive mental health treatment from the Wellbeing Center for their physical illness and lack of support from the family. Moreover, after childbirth, the physician referred the mothers to the Wellbeing Center based on the mental health examination. However, sudden release from the hospital is a reason for not receiving the services. A health worker mentioned the following:

Within the 42 days [postnatal period], mothers were physically sick to come. Those who are referred by the physician, sometimes get a sudden release from the hospital. Therefore, they do not come for mental health services.

KII, health assistant, age 33 years

##### Long Waiting Times

During the follow-up visit, antepartum and postpartum women have to wait in the ANC and PNC corner for taking services. Some patients were in a rush to receive the service, but it was not possible to give them an opportunity, as other patients were also on the waiting list. This may be due to the patient load and the ANC and PNC corners operating on a first-come, first-served basis. A pregnant mother mentioned the following:

I feel unsteady while waiting for [receiving] the mental health service. There are only three seats in the waiting room, and three mothers already waiting there to receive the service. Therefore, I and my husband have to stand up for around thirty to forty minutes which causes the irritation.

IDI, pregnant mother, age 18 years

## Discussion

### Principal Findings

Our research demonstrated that telemental health services through the Wellbeing Center are feasible, acceptable, useful, and highly needed among women. It has increased accessibility to mental health services to pregnant or postpartum women with a lower level of income and education. The health care providers felt a need for more staff with appropriate training to implement this intervention at the hospitals, who are lacking in Bangladesh. The women visiting the DHs have a higher level of depression and anxiety symptoms, demonstrating a critical need for mental health services. Our follow-up assessment scores on depression and anxiety symptoms after the first counseling sessions demonstrated a decrease in the average scores, which indicted the effectiveness of the Wellbeing Centers. The service beneficiaries recommended the intervention because of the positive attitude of the service providers; the services being free of cost, private, and confidential; and its sensitive naming. However, they also mentioned some barriers to receiving care from Wellbeing Centers, which included social stigma, postpartum illness, and long waiting times at the facility to use the service.

To the best of our knowledge, this is the first implementation research that assessed the WHO implementation outcome variables of a facility-based telemental health intervention, the Wellbeing Centers, for antepartum and postpartum women that is operated by the GoB. We have pioneered in proposing an implementation model for mental health care including a pool of trained psychologists and psychiatrists who provided counseling and medications for mental health issues through videoconferencing in the Wellbeing Centers at the public health facilities in Bangladesh.

Some public hospitals in Bangladesh had initiated telemedicine units as a part of their routine care system. However, telemental health services through Wellbeing Centers differ from general telemedicine in several ways. Telemental health often involves specialized platforms designed to provide psychological assessments, therapy, and psychiatric consultations. In contrast, telemedicine includes a broader range of remote clinical services, including primary care, specialist consultations, and follow-up visits. Telemental health is a specialized subset of telemedicine focusing on mental health. While both telemental health and telemedicine use videoconferencing, phone calls, and other digital communication tools, telemental health platforms are tailored to address the unique needs of mental health care, such as ensuring patient privacy and providing a comfortable environment for discussing sensitive issues. This specialization can lead to better patient engagement and satisfaction in mental health care compared to general telemedicine [47]. This type of service is expected to vary according to the differences in context and health systems. Our research focused on the implementation outcomes of telemental health care. This provides evidence to support the scale-up of telemental health care through Wellbeing Centers.

### Adoption and Acceptability

A significant proportion of Bangladeshi mothers have common mental health issues during the antenatal and postnatal period [4,48]. Most mental health care facilities are clustered around urban areas, while rural women do not have access to mental health care in Bangladesh. Since maternal mental distress among these rural mothers may cause adverse maternal and child health outcomes, appropriate interventions can address maternal mental distress among the Bangladeshi maternal population. Evidence supports the effectiveness of digital psychological interventions, especially in low- and middle-income countries where mental disorders contribute significantly to the global burden of disease [49,50]. Globally, these services offer private, personalized support, connecting disparities in mental health care for those facing challenges accessing traditional services or face-to-face services [49]. Global evidence emphasizes the role of telemental health service as an equivalent and effective method, particularly in delivering mental health services to remote areas with limited resources. A significant number of users in our study provided strong evidence on the adoptability and acceptability of the telemental health services through Wellbeing Centers in Bangladesh. Services through Wellbeing Centers minimized travel, offering cost-effective and accessible psychological and psychiatric services.

### Feasibility

The health care providers perceived that the telemental support through the Wellbeing Center is highly feasible. However, they also felt that the facilities lacked appropriately trained staff to maintain these centers. Banbury et al [51] stated that staff require comprehensive training to sustain and expand telehealth use in the facilities. These trainings should focus on knowledge, skills, and competencies in using telehealth as well as the broad factors of policies and understanding technologies to support the service providers [51]. Studies have discussed concerns around a lack of appropriate training to be able to conduct remote mental health care effectively and safely [52]. The government should ensure adequate training and supportive supervision for health providers (such as RMOs, physicians, gynecological consultants, and health workers) on mental health disorder assessments of pregnant and postpartum mothers, psychosocial services, as well as information and communication technology to maintain Wellbeing Center activities.

### Accessibility

In the context of Bangladesh, mental health services face challenges due to limited resources and a shortage of professionals, relying heavily on the NIMH in Dhaka [53,54]. The new mental health policy of Bangladesh prioritizes community-based services, with nongovernment organizations contributing scalable models, including telemental health initiatives [55,56]. Access challenges still persist despite some voluntary counseling platforms, due to their lack of visibility [57,58]. These programs are not also integrated into the government system. Despite progress and current provisions for service delivery, issues like low help-seeking, inadequate service delivery, and persistent stigma emphasize the need for telemental health services on a larger scale [59]. Integrating telemental health services with these services is essential for improved mental health care [58]. Wellbeing Centers increased perceived accessibility among antepartum and postpartum women. Perceived accessibility was significantly higher among lower socioeconomic groups and lower educated groups. One of the reasons for this could be the free-of-cost facilities at public hospitals that are near their location.

### Need

We looked into the need for well-being by looking at the depressive and anxiety symptoms prevalent among our targeted patients. Two-thirds of the women who sought services from the Wellbeing Center had moderate to severe indications of depression and anxiety. In addition, we observed a high level of anhedonia or lack of interest and tiredness among the women in the last 2 weeks. These highly prevalent symptoms indicated the need for Wellbeing Centers for the targeted mothers. El Sayed et al [60] reported that fatigue and anhedonia were prevalent and commonly reported in the post–COVID-19 period. A study by Costa et al [61] also reported that the prevalence of perinatal depression increased during the COVID-19 pandemic, which may be due to changes in the profile of specific depressive symptoms. Pearson et al [62] conducted a study where they reported that women experiencing anhedonic depressive symptoms during pregnancy had significantly larger systolic blood pressure responses toward infant distress than nondepressed pregnant women. Special attention should be given to anhedonia and fatigue-related symptoms of perinatal depression to ensure that they are adequately managed.

Among the other depressive symptoms, our finding that 3.8% (n=35) of patients experienced suicidal ideation is a significant concern, reinforcing the need for enhanced mental health interventions in Bangladesh. Scientific studies discussed the complexity of suicidal ideation, noting that it often goes unreported due to stigma and fear of judgment [63]. Suicidal ideation is influenced by a complex interplay of biological, psychological, and social factors. While some biomarkers have been identified to predict the risk of suicide, the underlying causes of suicide remain largely unclear. More research is needed to understand the root causes of suicidal ideation. Digital tools, such as mobile apps and telehealth services, can effectively monitor and reduce suicidal thoughts by providing real-time support [64]. Additionally, telehealth-supported decision-making has been found to significantly reduce suicidal ideation [65].

Our study also found that the level of worry and nervousness in the past 2 weeks was significantly higher among pregnant and postpartum women. More than half of the women experienced these symptoms of anxiety in the past 2 weeks almost every day. Tarafa et al [66] conducted a study in Ethiopia and assessed the factors associated with pregnancy-related anxiety among pregnant women attending ANC follow-up. Unwanted pregnancy, high perceived stress, young age, depression, low income, and poor social support were significantly associated with pregnancy-related anxiety. The overall prevalence of pregnancy-related anxiety in this study was slightly lower 32.7% [66]. It is worth noting that this study assessed only pregnancy-related anxiety while we included both pregnant and postpartum mothers. Appropriate intervention and focus are needed to address these worries and anxieties of pregnant women and postpartum mothers in Bangladesh through Wellbeing Center activities.

We report higher depression and anxiety and perceived accessibility among the women who took Wellbeing Center services at the district-level facilities compared to the subdistrict-level facilities. The reason can be regarded as an indirect effect of urbanization. A recent meta-analysis conducted by Cadman et al [67] assessed the influence of the urban environment in pregnancy and postpartum depression. Exposure to air pollution and road traffic congestion may increase maternal depression. The urban family structure, with a lack of family support, may also induce higher levels of depressive and anxiety symptoms in Bangladesh. A study conducted on adolescents researched screen-related sedentary behavior, finding that the use of social media caused 2 times higher depression among urban adolescents [68]. A large proportion of our users of Wellbeing Centers were adolescents. Therefore, urgent initiatives should be taken to control the spread of depression and anxiety among the urban population, especially for mothers. In summary, at the district-level facilities, the need for mental health services was higher among the antepartum and postpartum women, which necessitated a strengthened focus on providing equitable services through the Wellbeing Centers in Bangladesh.

### Usefulness

While we found the Wellbeing Centers to be feasible and acceptable, our study also indicated small improvements during the follow-up counseling sessions for the targeted patients. A review by Hilty et al [34] reported that telemental health is effective for diagnosis and assessment across many populations, including adults, children, older people, and people of different ethnicities, and for disorders in home and facility settings. This review urged that more research should be conducted on service models, specific disorders, issues regarding culture and language, and cost. Ensuring follow-up visits after the first counseling session is important for the sustained impact of telemental health services. However, we have not observed many women taking follow-up services in our Wellbeing Centers. Special initiatives such as follow-up phone calls or reminders should be ensured to increase follow-up sessions when needed and recommended.

### Experience, Perception, and Expectation

Our research explored the experiences and perceptions regarding the Wellbeing Center activities, which revealed several strengths of and barriers to using these services. The users acknowledged the positive attitude and patience of the counselors during the service sessions. In Bangladesh, in-person psychiatric services are expensive and may not be affordable for underprivileged women. The Wellbeing Center services were completely free of cost, which attracted the targeted patients. Furthermore, these Wellbeing Centers ensured the privacy and confidentiality of the patients while providing psychological or psychiatric support. Many women were comfortable while expressing their problems and mental health issues, as they were reassured by this confidentiality. Finally, the women praised the naming of the service, which also addressed the stigma around mental health. The women also mentioned that existing stigmas and taboos on seeking mental health services prevented them from seeking care. However, almost all users of the Wellbeing Centers agreed that these services were acceptable to them. Further research is necessary to understand the actual state of stigma in the community regarding mental health care seeking.

Our quantitative data suggested that more women during pregnancy used Wellbeing Center services compared to postpartum women. Our qualitative finding echoed this finding as women reported postpartum illnesses were a barrier for them to seeking mental health care. Finally, few women mentioned that the long waiting time repelled them to receive services from the Wellbeing Centers. With the high demand and popularity, the system experienced a high patient load and struggled to satisfy all patients with timely services.

### Comparison With Prior Studies

Recent studies on telemental health interventions have shown promising results, particularly in the context of the COVID-19 pandemic, which accelerated the adoption of remote mental health care. Research indicates that telemental health, including videoconferencing and phone-based therapy, is generally as effective as in-person care for a variety of mental health conditions [47]. Our research found similar results in terms of feasibility, utility, and effectiveness. A recent systematic review on implementation strategies for telemental health published in 2023 by Appleton et al [52] highlighted that telemental health can improve access to care, especially for individuals in remote or resource-limited areas. Our study echoed them in terms of the role of telemental health support in increasing the accessibility of mental health care in Bangladesh. While most studies on telemental health mentioned positive outcomes, one of the meta-analyses on mobile phone–based telemental health interventions by Goldberg et al [69] suggested that the effectiveness of these interventions can vary based on the specific mental health condition and the technology used. Our study could not compare the efficacy of the Wellbeing Centers compared to other phone-based or community-based methods. We recommend further research and trials to understand the actual benefits of using Wellbeing Centers compared to other types of services.

### Strengths

Our study has several strengths, based on which we have identified the major findings discussed earlier. This study was conducted in 6 facilities, which provided us a reasonably large sample of pregnant and postpartum women attending antenatal and postpartum care. It involved a rigorous analysis using WHO-guided implementation variable, which ensured standardization with other global implementation research on telemental health services. Our intervention was provided through the government system; therefore, these findings will be valuable for the GoB for scaling up the intervention to a higher number of facilities. This paper focused on the pregnant and postpartum women. However, the services are also provided to general patients. Therefore, findings might provide additional evidence while assessing the implementation outcomes for general patients.

### Limitations

We also acknowledge some limitations of our study. First, this study was conceptualized based on the WHO framework and implementation outcome variables. The WHO’s implementation outcome variables may have limited the opportunities of capturing other potential outcome variables that may be important for assessing implementation aspects of the Wellbeing Centers. Second, we acknowledge the fact that we did not randomly select our demonstration sites for Wellbeing Centers. Therefore, the result may not be generalizable to the whole country. In Bangladesh, there are regional variations that have an impact on access to health care as well as service quality. Our selected sites reasonably capture the variations of the service provision and quality. Third, we also acknowledge that we have selected the DHs based on their functionality. The inclusion of low-performing districts could make our results more generalizable. However, this was not possible because of implementation cost challenges. Fourth, in this analysis, we could not assess the rate of adoption of the Wellbeing Center services, as we could not capture the true denominator of how many women needed mental health support among those seeking ANC or PNC from the facilities. Finally, this analysis only assessed symptoms of depression and anxiety as mental health issue. Other disorders could also have been included to strengthen this study.

### Conclusions

This implementation research study demonstrated the feasibility, acceptability, and usefulness of introducing the telemental health service Wellbeing Centers for antepartum and postpartum women at Bangladeshi facilities. We are confident in our conclusions, as we saw the services increased the perceived accessibility of mental health services with minimal influence from other factors. Appropriate staff training is required to maintain these centers. We recommend that psychologists and psychiatrists have patience and a positive attitude while maintaining the privacy of patients during the scale-up of the model. We also recommend future studies on cost-effectiveness and postimplementation follow-up to evaluate the sustainability, effectiveness, and impact over a longer time period. The experiences and learnings from this implementation research can support generating evidence-based decisions related to the introduction and scaling-up of the Wellbeing Centers in Bangladesh and other low- and middle-income countries.

## Appendix

Multimedia Appendix 1 Quantitative data collection tools to assess depression, anxiety, adoption, accessibility, acceptability, and usefulness for antenatal and postnatal women.

Multimedia Appendix 2 Data collection tool for feasibility assessment among the health facility managers.

Multimedia Appendix 3 Interview guideline for in-depth interviews with antenatal and postnatal women to explore their experiences, perceptions, and expectations regarding the telemental health counseling.

Multimedia Appendix 4 Interview guideline for key informant interviews with the counselors and health care providers to explore their experiences, perceptions, and expectations regarding the telemental health counseling.

Multimedia Appendix 5 Flowchart of the selected antepartum and postpartum women.

Multimedia Appendix 6 Proportion of users who received follow-up counseling.

## Abbreviations

ANC: antenatal care
aOR: adjusted odds ratio
DH: district hospital
GAD-7: Generalized Anxiety Disorder
GoB: government of Bangladesh
icddr,b: International Centre for Diarrhoeal Disease Research, Bangladesh
IDI: in-depth interview
KII: key informant interview
NCDC: Non-Communicable Disease Control
NIMH: National Institute of Mental Health
PHQ-9: Patient Health Questionnaire-9
PNC: postnatal care
RMO: resident medical officer
UHC: upazila health complex
WHO: World Health Organization

## References

[1] Jones I, Chandra PS, Dazzan P, Howard LM (2014). Bipolar disorder, affective psychosis, and schizophrenia in pregnancy and the post-partum period. Lancet.

[2] Maternal mental health. World Health Organization.

[3] Nguyen PH, Saha KK, Ali D, Menon P, Manohar S, Mai LT, Rawat R, Ruel MT (2014). Maternal mental health is associated with child undernutrition and illness in Bangladesh, Vietnam and Ethiopia. Public Health Nutr.

[4] Nasreen HE, Kabir ZN, Forsell Y, Edhborg M (2011). Prevalence and associated factors of depressive and anxiety symptoms during pregnancy: a population based study in rural Bangladesh. BMC Womens Health.

[5] Edhborg M, Nasreen H, Kabir ZN (2011). Impact of postpartum depressive and anxiety symptoms on mothers' emotional tie to their infants 2-3 months postpartum: a population-based study from rural Bangladesh. Arch Womens Ment Health.

[6] Hossain SJ, Roy BR, Hossain AT, Mehrin F, Tipu SMMU, Tofail F, Arifeen SE, Tran T, Fisher J, Hamadani J (2020). Prevalence of maternal postpartum depression, health-seeking behavior and out of pocket payment for physical illness and cost coping mechanism of the poor families in Bangladesh: a rural community-based study. Int J Environ Res Public Health.

[7] Saccone G, Florio A, Aiello F, Venturella R, De Angelis MC, Locci M, Bifulco G, Zullo F, Di Spiezio Sardo A (2020). Psychological impact of coronavirus disease 2019 in pregnant women. Am J Obstet Gynecol.

[8] Hamadani JD, Hasan MI, Baldi AJ, Hossain SJ, Shiraji S, Bhuiyan MSA, Mehrin SF, Fisher J, Tofail F, Tipu SMMU, Grantham-McGregor S, Biggs B, Braat S, Pasricha S (2020). Immediate impact of stay-at-home orders to control COVID-19 transmission on socioeconomic conditions, food insecurity, mental health, and intimate partner violence in Bangladeshi women and their families: an interrupted time series. Lancet Glob Health.

[9] Fekadu Dadi A, Miller ER, Mwanri L (2020). Antenatal depression and its association with adverse birth outcomes in low and middle-income countries: a systematic review and meta-analysis. PLoS One.

[10] Dadi AF, Miller ER, Bisetegn TA, Mwanri L (2020). Global burden of antenatal depression and its association with adverse birth outcomes: an umbrella review. BMC Public Health.

[11] Dadi AF, Miller ER, Mwanri L (2020). Postnatal depression and its association with adverse infant health outcomes in low- and middle-income countries: a systematic review and meta-analysis. BMC Pregnancy Childbirth.

[12] Sahoo S, Gill G, Sikka P, Nehra R (2023). Antenatal depression and anxiety in Indian women: a systematic review. Ind Psychiatry J.

[13] Joshi D, Shrestha S, Shrestha N (2019). Understanding the antepartum depressive symptoms and its risk factors among the pregnant women visiting public health facilities of Nepal. PLoS One.

[14] Gelaye B, Rondon MB, Araya R, Williams MA (2016). Epidemiology of maternal depression, risk factors, and child outcomes in low-income and middle-income countries. Lancet Psychiatry.

[15] Rusner M, Berg M, Begley C (2016). Bipolar disorder in pregnancy and childbirth: a systematic review of outcomes. BMC Pregnancy Childbirth.

[16] Vigod SN, Kurdyak PA, Dennis CL, Gruneir A, Newman A, Seeman MV, Rochon PA, Anderson GM, Grigoriadis S, Ray JG (2014). Maternal and newborn outcomes among women with schizophrenia: a retrospective population-based cohort study. BJOG.

[17] McAllister-Williams RH, Baldwin DS, Cantwell R, Easter A, Gilvarry E, Glover V, Green L, Gregoire A, Howard LM, Jones I, Khalifeh H, Lingford-Hughes A, McDonald E, Micali N, Pariante CM, Peters L, Roberts A, Smith NC, Taylor D, Wieck A, Yates LM, Young AH (2017). British Association for Psychopharmacology consensus guidance on the use of psychotropic medication preconception, in pregnancy and postpartum 2017. J Psychopharmacol.

[18] Laursen TM, Munk-Olsen T (2010). Reproductive patterns in psychotic patients. Schizophr Res.

[19] Nasreen HE, Pasi HB, Rifin SM, Aris MAM, Rahman JA, Rus RM, Edhborg M (2019). Impact of maternal antepartum depressive and anxiety symptoms on birth outcomes and mode of delivery: a prospective cohort study in east and west coasts of Malaysia. BMC Pregnancy Childbirth.

[20] Slomian J, Honvo G, Emonts P, Reginster J, Bruyère O (2019). Consequences of maternal postpartum depression: a systematic review of maternal and infant outcomes. Womens Health (Lond).

[21] Howard LM, Khalifeh H (2020). Perinatal mental health: a review of progress and challenges. World Psychiatry.

[22] Alves SP, Costa T, Ribeiro I, Néné M, Sequeira C (2023). Perinatal mental health counselling programme: a scoping review. Patient Educ Couns.

[23] (2020). Bangladesh: WHO special initiative for mental health situational assessment. World Health Organization.

[24] Khan MM, Rahman SMT, AnjumIslam ST (2021). The use of telemedicine in Bangladesh during COVID-19 pandemic. E-Health Telecommun Syst Netw.

[25] Huang L, Zhao Y, Qiang C, Fan B (2018). Is cognitive behavioral therapy a better choice for women with postnatal depression? A systematic review and meta-analysis. PLoS One.

[26] Barrera AZ, Wickham RE, Muñoz RF (2015). Online prevention of postpartum depression for Spanish- and English-speaking pregnant women: a pilot randomized controlled trial. Internet Interv.

[27] Fu Z, Burger H, Arjadi R, Bockting CLH (2020). Effectiveness of digital psychological interventions for mental health problems in low-income and middle-income countries: a systematic review and meta-analysis. Lancet Psychiatry.

[28] Wang L, Tsai H, Chen Y, Jhang J, Wu P, Huang Y, Lee M, Chen L, Yu W, Chiang M (2024). A preliminary study of the effectiveness of video visitation on depression and stress in mothers with preterm infants during the pandemic. Pediatr Neonatol.

[29] Fernandez E, Woldgabreal Y, Day A, Pham T, Gleich B, Aboujaoude E (2021). Live psychotherapy by video versus in-person: a meta-analysis of efficacy and its relationship to types and targets of treatment. Clin Psychol Psychother.

[30] Zhao L, Chen J, Lan L, Deng N, Liao Y, Yue L, Chen I, Wen SW, Xie R (2021). Effectiveness of telehealth interventions for women with postpartum depression: systematic review and meta-analysis. JMIR Mhealth Uhealth.

[31] Loughnan SA, Joubert AE, Grierson A, Andrews G, Newby JM (2019). Internet-delivered psychological interventions for clinical anxiety and depression in perinatal women: a systematic review and meta-analysis. Arch Womens Ment Health.

[32] Mu TY, Li YH, Xu RX, Chen J, Wang YY, Shen CZ (2021). Internet-based interventions for postpartum depression: a systematic review and meta-analysis. Nurs Open.

[33] Huang S, Wang Y, Li G, Hall BJ, Nyman TJ (2024). Digital mental health interventions for alleviating depression and anxiety during psychotherapy waiting lists: systematic review. JMIR Ment Health.

[34] Hilty DM, Ferrer DC, Parish MB, Johnston B, Callahan EJ, Yellowlees PM (2013). The effectiveness of telemental health: a 2013 review. Telemed J E Health.

[35] Bulkes NZ, Davis K, Kay B, Riemann BC (2022). Comparing efficacy of telehealth to in-person mental health care in intensive-treatment-seeking adults. J Psychiatr Res.

[36] Ackerman M, Greenwald E, Noulas P, Ahn C (2021). Patient satisfaction with and use of telemental health services in the perinatal period: a survey study. Psychiatr Q.

[37] Kılıç ST, Taşgıt A (2023). Sociodemographic factors affecting depression-anxiety-stress levels and coping strategies of parents with babies treated in neonatal intensive care units during the COVID-19 pandemic. J Neonatal Nurs.

[38] Dhira TA, Rahman MA, Sarker AR, Mehareen J (2021). Validity and reliability of the Generalized Anxiety Disorder-7 (GAD-7) among university students of Bangladesh. PLoS One.

[39] Kamrul-Hasan ABM, Hannan MA, Asaduzzaman M, Rahman MM, Alam MS, Amin MN, Kabir MR, Chanda PK, Jannat N, Haque MZ, Banik SR, Hasan MJ, Selim S (2022). Prevalence and predictors of diabetes distress among adults with type 2 diabetes mellitus: a facility-based cross-sectional study of Bangladesh. BMC Endocr Disord.

[40] Naher R, Rabby M, Sharif F (2021). Validation of Patient Health Questionnaire-9 for assessing depression of adults in Bangladesh. Dhaka Univ J Biol Sci.

[41] Shih SCS, Chan ASM, Yeung EYY, Tsang AMY, Chiu RLP, Chu MHW, Poon MYC (2021). Psychometric properties of Chinese version of work and social adjustment scale for outpatients with common mental disorders: classical test theory and Rasch analysis. East Asian Arch Psychiatry.

[42] Peters DH, Tran NT, Adam T (2013). Implementation research in health: a practical guide. World Health Organization.

[43] Kroenke K, Spitzer RL, Williams JB (2001). The PHQ-9: validity of a brief depression severity measure. J Gen Intern Med.

[44] Spitzer RL, Kroenke K, Williams JBW, Löwe B (2006). A brief measure for assessing generalized anxiety disorder: the GAD-7. Arch Intern Med.

[45] Braun V, Clarke V (2006). Using thematic analysis in psychology. Qual Res Psychol.

[46] Thomas DR (2006). A general inductive approach for analyzing qualitative evaluation data. Am J Eval.

[47] Sugarman DE, Busch AB (2023). Telemental health for clinical assessment and treatment. BMJ.

[48] Azad R, Fahmi R, Shrestha S, Joshi H, Hasan M, Khan ANS, Chowdhury MAK, Arifeen SE, Billah SM (2019). Prevalence and risk factors of postpartum depression within one year after birth in urban slums of Dhaka, Bangladesh. PLoS One.

[49] Mishkind MC (2021). How telemental health delivered to non-traditional locations helped prepare for responses to COVID-19. Mhealth.

[50] O'Brien M, McNicholas F (2020). The use of telepsychiatry during COVID-19 and beyond. Ir J Psychol Med.

[51] Banbury A, Taylor ML, Gray LC, Reid N, Smith AC (2023). Sustaining and expanding telehealth activity: training requirements for Australian residential aged care front-line staff. PEC Innov.

[52] Appleton R, Barnett P, Vera San Juan N, Tuudah E, Lyons N, Parker J, Roxburgh E, Spyridonidis S, Tamworth M, Worden M, Yilmaz M, Sevdalis N, Lloyd-Evans B, Needle JJ, Johnson S (2023). Implementation strategies for telemental health: a systematic review. BMC Health Serv Res.

[53] Hasan MT, Anwar T, Christopher E, Hossain S, Hossain MM, Koly KN, Saif-Ur-Rahman KM, Ahmed HU, Arman N, Hossain SW (2021). The current state of mental healthcare in Bangladesh: part 1—an updated country profile. BJPsych Int.

[54] Islam A (2015). Mental health and the health system in Bangladesh: situation analysis of a neglected domain. AJPN.

[55] Strengthening inclusive community based mental health support. Center for Disability in Development.

[56] Sharmin F, Chowdhury M (2023). Initiating mental health and psychosocial support at the community level in Bangladesh. The Business Standards.

[57] (2016). Esho Nije Kori—Best Mental Health Organization in Bangladesh.

[58] Mental Health Service Directory 2023. ADD International Bangladesh.

[59] Hossain MD, Ahmed HU, Chowdhury WA, Niessen LW, Alam DS (2014). Mental disorders in Bangladesh: a systematic review. BMC Psychiatry.

[60] El Sayed S, Shokry D, Gomaa SM (2021). Post-COVID-19 fatigue and anhedonia: a cross-sectional study and their correlation to post-recovery period. Neuropsychopharmacol Rep.

[61] Costa R, Pinto TM, Conde A, Mesquita A, Motrico E, Figueiredo B (2023). Women's perinatal depression: anhedonia-related symptoms have increased in the COVID-19 pandemic. Gen Hosp Psychiatry.

[62] Pearson RM, Lightman SL, Evans J (2012). Symptoms of depression during pregnancy are associated with increased systolic blood pressure responses towards infant distress. Arch Womens Ment Health.

[63] Bruen AJ, Wall A, Haines-Delmont A, Perkins E (2020). Exploring suicidal ideation using an innovative mobile app-strength within me: the usability and acceptability of setting up a trial involving mobile technology and mental health service users. JMIR Ment Health.

[64] Toscos T, Coupe A, Flanagan M, Drouin M, Carpenter M, Reining L, Roebuck A, Mirro MJ (2019). Teens using screens for help: impact of suicidal ideation, anxiety, and depression levels on youth preferences for telemental health resources. JMIR Ment Health.

[65] O'Callaghan E, Mahrer N, Belanger HG, Sullivan S, Lee C, Gupta CT, Winsberg M (2022). Telehealth-supported decision-making psychiatric care for suicidal ideation: longitudinal observational study. JMIR Form Res.

[66] Tarafa H, Alemayehu Y, Nigussie M (2022). Factors associated with pregnancy-related anxiety among pregnant women attending antenatal care follow-up at Bedelle general hospital and Metu Karl comprehensive specialized hospital, Southwest Ethiopia. Front Psychiatry.

[67] Cadman T, Strandberg-Larsen K, Calas L, Christiansen M, Culpin I, Dadvand P, de Castro M, Foraster M, Fossati S, Guxens M, Harris JR, Hillegers M, Jaddoe V, Lee Y, Lepeule J, El Marroun H, Maule M, McEachen R, Moccia C, Nader J, Nieuwenhuijsen M, Nybo Andersen AM, Pearson R, Swertz M, Vafeiadi M, Vrijheid M, Wright J, Lawlor DA, Pedersen M (2024). Urban environment in pregnancy and postpartum depression: an individual participant data meta-analysis of 12 European birth cohorts. Environ Int.

[68] Anjum A, Hossain S, Sikder T, Uddin ME, Rahim DA (2022). Investigating the prevalence of and factors associated with depressive symptoms among urban and semi-urban school adolescents in Bangladesh: a pilot study. Int Health.

[69] Goldberg SB, Lam SU, Simonsson O, Torous J, Sun S (2022). Mobile phone-based interventions for mental health: a systematic meta-review of 14 meta-analyses of randomized controlled trials. PLOS Digit Health.

