# Supplementary material for: Enhancing Access to Mental Health Services for Antepartum and Postpartum Women Through Telemental Health Services at Wellbeing Centers in Selected Health Facilities in Bangladesh: Implementation Research
Source: JMIR Pediatr Parent. 2025 Jan 3;8:e65912. doi: 10.2196/65912 (PMC11748442; doi:10.2196/65912)
Supplement: Multimedia Appendix 1 [file pediatrics_v8i1e65912_app1.docx]

| Participant’s ID……………………………………………………………….............………………  Name of the participant…………………………………………………………………………………  Address of the participant………………………………………………………………………………  Phone number of the participant………………………………………………………………..... | | | | | |
| --- | --- | --- | --- | --- | --- |
| **Section A: Patient Health Questionnaires** | | | | | |
| **SL** | **Variables** | **Not at all** | **Several days** | **More than half the days** | **Nearly every day** |
|  | Little interest or pleasure in doing things | 0 | 1 | 2 | 3 |
|  | Feeling down, depressed, or hopeless | 0 | 1 | 2 | 3 |
|  | Trouble falling or staying asleep, or sleeping too much | 0 | 1 | 2 | 3 |
|  | Feeling tired or having little energy | 0 | 1 | 2 | 3 |
|  | Poor appetite or overeating | 0 | 1 | 2 | 3 |
|  | Feeling bad about yourself — or that you are a failure or  have let yourself or your family down | 0 | 1 | 2 | 3 |
|  | Trouble concentrating on things, such as reading the newspaper or watching television | 0 | 1 | 2 | 3 |
|  | Moving or speaking so slowly that other people could have noticed? Or the opposite — being so fidgety or restless  that you have been moving around a lot more than usual | 0 | 1 | 2 | 3 |
|  | Thoughts that you would be better off dead or of hurting yourself in some way | 0 | 1 | 2 | 3 |
| **Column totals** | | |  |  |  |
| **Total** | | |  | | |

| **Section B: Generalised Anxiety Disorder** | | | | | |
| --- | --- | --- | --- | --- | --- |
| **SL** | **Variables** | **Not at all** | **Several days** | **More than half the days** | **Nearly every day** |
|  | Feeling nervous, anxious, or on edge | 0 | 1 | 2 | 3 |
|  | Not being able to stop or control worrying | 0 | 1 | 2 | 3 |
|  | Worrying too much about different things | 0 | 1 | 2 | 3 |
|  | Trouble relaxing | 0 | 1 | 2 | 3 |
|  | Being so restless that it is hard to sit still | 0 | 1 | 2 | 3 |
|  | Becoming easily annoyed or irritable | 0 | 1 | 2 | 3 |
|  | Feeling afraid, as if something awful might happen | 0 | 1 | 2 | 3 |
| **Column totals** | | |  |  |  |
| **Total** | | |  | | |

| **Section C: Pregnancy information** | | | | | | | |
| --- | --- | --- | --- | --- | --- | --- | --- |
| **SL** | **Characteristic** |  | | **Tick here** | | **Skip** | |
|  | Weeks Pregnant | __________________ (running week) | | | |  | |
|  | Weeks of post-partum | __________________ (running week) | | | |  | |
|  | Number of children | __________________ (Number) | | | |  | |
|  | Mode of delivery for last pregnancy | N/A (First pregnancy) | |  | |  | |
|  |  | Vaginal delivery | |  | |  | |
|  |  | C-section | |  | |  | |
|  |  | Abortion | |  | |  | |
|  | History of miscarriage | Yes | |  | |  | |
|  |  | No | |  | |  | |
|  | History of still birth | Yes | |  | |  | |
|  |  | No | |  | |  | |
|  | Presence of chronic disease   1. Chronic Fever 2. Injuries / Disability 3. Chronic Heart Disease 4. Respiratory Diseases/ Asthma/Bronchitis/Chronic Obstetric Pulmonary Disease (COPD) 5. Altered bowel habit 6. Gastric/ ulcer 7. Blood pressure related problem (hypertension or hypertension) 8. Arthritis/ Rheumatism 9. Skin problem 10. Diabetes 11. Cancer 12. kidney Diseases 13. Liver Diseases 14. Mental Health 15. Paralysis/neurological disorder 16. Ear/ENT problem 17. Eye problem 18. Other (specify)   98 = DON’T KNOW | Interest code [insert multiple code with comma, if necessary]   \|  \| \| --- \| | |  | |  | |
| **Section D: Demographic information** | | | | | | | |
|  | Age | | __________________ (In year) | | | |  |
|  | Religion | | Islam | | 1 | |  |
|  |  |  | Hindu | | 2 | |  |
|  |  |  | Christian | | 3 | |  |
|  |  |  | Buddha | | 4 | |  |
|  |  |  | Others (specify)  _____________ | | 5 | |  |
|  | Current marital status | | Married (or in a domestic partnership) | | 1 | |  |
|  |  |  | Divorced | | 2 | |  |
|  |  |  | Other (specify)  _____________ | | 3 | |  |
|  | Type of marriage | | Arranged | | 1 | |  |
|  |  |  | Mutual consent | | 2 | |  |
|  | Employment status of the mother | | Full-time employed | | 1 | |  |
|  |  |  | Homemaker | | 2 | |  |
|  |  |  | Unable to work due to disability | | 3 | |  |
|  | Total years of education | | __________________ (In year) | | | |  |
|  | Level of education | | No education | | 1 | |  |
|  |  |  | Primary incomplete | | 2 | |  |
|  |  |  | Primary complete | | 3 | |  |
|  |  |  | Secondary incomplete | | 4 | |  |
|  |  |  | Secondary complete or higher | | 5 | |  |
|  | Level of education of husband | | No education | | 1 | |  |
|  |  |  | Primary incomplete | | 2 | |  |
|  |  |  | Primary complete | | 3 | |  |
|  |  |  | Secondary incomplete | | 4 | |  |
|  |  |  | Secondary complete or higher | | 5 | |  |
|  |  |  | Not applicable | | 6 | |  |
|  | Employment status of the husband | | Housewife | | 1 | |  |
|  |  |  | Civil servant | | 2 | |  |
|  |  |  | Worker | | 3 | |  |
|  |  |  | Self-employed | | 4 | |  |
|  |  |  | Employed in abroad | | 5 | |  |
|  |  |  | Not applicable | | 6 | |  |
|  | Family income | | __________________ (In Tk) | | | |  |

|  | **Section E: Care related information** | | | |
| --- | --- | --- | --- | --- |
| **SL** | **Characteristic** | **Options** | | **Skip** |
|  | Distance from home to this district hospital | __________________ (In Kilometre) | |  |
|  |  | I don’t know……………………… | |  |
|  | Travel time from home to this district hospital | __________________ (In Minute) | |  |
|  |  | I don’t know……………………… | |  |
|  | Total number of telehealth visits during pregnancy and the first-year post-partum including current visit | __________________ (Number) | |  |
|  | Type of treatment for which telehealth services were used in current visit | Medication management |  |  |
|  |  | Psychotherapy |  |  |
|  |  | Combination of above |  |  |

| **Section F: Acceptability and user experience** | | | | |
| --- | --- | --- | --- | --- |
| **SL** | **Characteristic** | **Options** |  | **Skip** |
|  | It was simple to use the tele-mental health system in well-being corner | Strongly disagree | 1 |  |
|  |  | Disagree | 2 |  |
|  |  | Neither agree nor disagree | 3 |  |
|  |  | Agree | 4 |  |
|  |  | Strongly agree | 5 |  |
|  | When I experienced a technical difficulty, someone was able to assist me and resolve the problem | Strongly disagree | 1 |  |
|  |  | Disagree | 2 |  |
|  |  | Neither agree nor disagree | 3 |  |
|  |  | Agree | 4 |  |
|  |  | Strongly agree | 5 |  |
|  | It was easy to learn how to use the tele-mental health system in well-being corner | Strongly disagree | 1 |  |
|  |  | Disagree | 2 |  |
|  |  | Neither agree nor disagree | 3 |  |
|  |  | Agree | 4 |  |
|  |  | Strongly agree | 5 |  |
|  | I could easily talk to the counsellor using the tele-mental health system in well-being corner | Strongly disagree | 1 |  |
|  |  | Disagree | 2 |  |
|  |  | Neither agree nor disagree | 3 |  |
|  |  | Agree | 4 |  |
|  |  | Strongly agree | 5 |  |
|  | I could hear the clinician clearly using the tele-mental health system in well-being corner | Strongly disagree | 1 |  |
|  |  | Disagree | 2 |  |
|  |  | Neither agree nor disagree | 3 |  |
|  |  | Agree | 4 |  |
|  |  | Strongly agree | 5 |  |
|  | I felt I was able to express myself effectively to the counsellor by this system | Strongly disagree | 1 |  |
|  |  | Disagree | 2 |  |
|  |  | Neither agree nor disagree | 3 |  |
|  |  | Agree | 4 |  |
|  |  | Strongly agree | 5 |  |
|  | I felt that the counsellor paid attention to me | Strongly disagree | 1 |  |
|  |  | Disagree | 2 |  |
|  |  | Neither agree nor disagree | 3 |  |
|  |  | Agree | 4 |  |
|  |  | Strongly agree | 5 |  |
|  | I felt comfortable communicating with the counsellor using the telehealth system | Strongly disagree | 1 |  |
|  |  | Disagree | 2 |  |
|  |  | Neither agree nor disagree | 3 |  |
|  |  | Agree | 4 |  |
|  |  | Strongly agree | 5 |  |
|  | I felt comfortable with the privacy of the well-being corner | Strongly disagree | 1 |  |
|  |  | Disagree | 2 |  |
|  |  | Neither agree nor disagree | 3 |  |
|  |  | Agree | 4 |  |
|  |  | Strongly agree | 5 |  |
|  | I believe I feel better after using this tele-mental health system in well-being corner | Strongly disagree | 1 |  |
|  |  | Disagree | 2 |  |
|  |  | Neither agree nor disagree | 3 |  |
|  |  | Agree | 4 |  |
|  |  | Strongly agree | 5 |  |
|  | I like using the tele-mental health system in well-being corner | Strongly disagree | 1 |  |
|  |  | Disagree | 2 |  |
|  |  | Neither agree nor disagree | 3 |  |
|  |  | Agree | 4 |  |
|  |  | Strongly agree | 5 |  |
|  | Overall, I am satisfied with this telehealth system | Strongly disagree | 1 |  |
|  |  | Disagree | 2 |  |
|  |  | Neither agree nor disagree | 3 |  |
|  |  | Agree | 4 |  |
|  |  | Strongly agree | 5 |  |

| **Section G: Usefulness** | | | | |
| --- | --- | --- | --- | --- |
| **SL** | **Characteristic** | **Options** |  | **Skip** |
|  | Well-being corner enabled me to access mental health services | Strongly disagree | 1 |  |
|  |  | Disagree | 2 |  |
|  |  | Neither agree nor disagree | 3 |  |
|  |  | Agree | 4 |  |
|  |  | Strongly agree | 5 |  |
|  | Using the tele-mental health system, I could see the clinician as well as if we met in person | Strongly disagree | 1 |  |
|  |  | Disagree | 2 |  |
|  |  | Neither agree nor disagree | 3 |  |
|  |  | Agree | 4 |  |
|  |  | Strongly agree | 5 |  |
|  | This well-being corner is able to provide everything I would want for my mental health | Strongly disagree | 1 |  |
|  |  | Disagree | 2 |  |
|  |  | Neither agree nor disagree | 3 |  |
|  |  | Agree | 4 |  |
|  |  | Strongly agree | 5 |  |

| **Section H: Utility** | | | | |
| --- | --- | --- | --- | --- |
|  | I will use telehealth services again if I need | Strongly disagree | 1 |  |
|  |  | Disagree | 2 |  |
|  |  | Neither agree nor disagree | 3 |  |
|  |  | Agree | 4 |  |
|  |  | Strongly agree | 5 |  |
|  | I would recommend this service to a friend or family member | Strongly disagree | 1 |  |
|  |  | Disagree | 2 |  |
|  |  | Neither agree nor disagree | 3 |  |
|  |  | Agree | 4 |  |
|  |  | Strongly agree | 5 |  |
