# Supplementary material for: Enhancing Access to Mental Health Services for Antepartum and Postpartum Women Through Telemental Health Services at Wellbeing Centers in Selected Health Facilities in Bangladesh: Implementation Research
Source: JMIR Pediatr Parent. 2025 Jan 3;8:e65912. doi: 10.2196/65912 (PMC11748442; doi:10.2196/65912)
Supplement: Multimedia Appendix 2 [file pediatrics_v8i1e65912_app2.docx]

| **Participant’s ID**  **Name**  **Designation**  **Ward**  **Years worked as health provider**  **Years/months worked in this facility**  **Years/months worked in this ward**  **Highest Education (in grade and degree)**  **Age**  **Gender**  **Phone number** | | | | |
| --- | --- | --- | --- | --- |
| **Section A: Feasibility** | | | | |
|  | The well-being corner fit with the existing infrastructure of this facility | Strongly disagree | 1 |  |
|  |  | Disagree | 2 |  |
|  |  | Neither agree nor disagree | 3 |  |
|  |  | Agree | 4 |  |
|  |  | Strongly agree | 5 |  |
|  | The well-being corner fit with the existing policy and plan of this facility | Strongly disagree | 1 |  |
|  |  | Disagree | 2 |  |
|  |  | Neither agree nor disagree | 3 |  |
|  |  | Agree | 4 |  |
|  |  | Strongly agree | 5 |  |
|  | This well-being corner preserves the human rights and privacy of the participants | Strongly disagree | 1 |  |
|  |  | Disagree | 2 |  |
|  |  | Neither agree nor disagree | 3 |  |
|  |  | Agree | 4 |  |
|  |  | Strongly agree | 5 |  |
|  | This well-being corner will meet the demand for mental healthcare among the locals | Strongly disagree | 1 |  |
|  |  | Disagree | 2 |  |
|  |  | Neither agree nor disagree | 3 |  |
|  |  | Agree | 4 |  |
|  |  | Strongly agree | 5 |  |
|  | Clients are being benefited by the well-being corner | Strongly disagree | 1 |  |
|  |  | Disagree | 2 |  |
|  |  | Neither agree nor disagree | 3 |  |
|  |  | Agree | 4 |  |
|  |  | Strongly agree | 5 |  |
|  | This well-being corner will raise awareness among the locals about mental well-being | Strongly disagree | 1 |  |
|  |  | Disagree | 2 |  |
|  |  | Neither agree nor disagree | 3 |  |
|  |  | Agree | 4 |  |
|  |  | Strongly agree | 5 |  |
|  | This facility has adequate human resources to carry on the well-being corner | Strongly disagree | 1 |  |
|  |  | Disagree | 2 |  |
|  |  | Neither agree nor disagree | 3 |  |
|  |  | Agree | 4 |  |
|  |  | Strongly agree | 5 |  |
|  | The healthcare professionals in this facility are adequately skilled of carrying out this well-being corner | Strongly disagree | 1 |  |
|  |  | Disagree | 2 |  |
|  |  | Neither agree nor disagree | 3 |  |
|  |  | Agree | 4 |  |
|  |  | Strongly agree | 5 |  |
|  | Continuous funding can be allocated to carry-out this well-being corner in this facility | Strongly disagree | 1 |  |
|  |  | Disagree | 2 |  |
|  |  | Neither agree nor disagree | 3 |  |
|  |  | Agree | 4 |  |
|  |  | Strongly agree | 5 |  |
|  | People will continue to utilise this well-being corner for their mental health well-being | Strongly disagree | 1 |  |
|  |  | Disagree | 2 |  |
|  |  | Neither agree nor disagree | 3 |  |
|  |  | Agree | 4 |  |
|  |  | Strongly agree | 5 |  |
|  | I think this well-being corner can be implemented other health facilities in Bangladesh | Strongly disagree | 1 |  |
|  |  | Disagree | 2 |  |
|  |  | Neither agree nor disagree | 3 |  |
|  |  | Agree | 4 |  |
|  |  | Strongly agree | 5 |  |
