# Supplementary material for: Enhancing Access to Mental Health Services for Antepartum and Postpartum Women Through Telemental Health Services at Wellbeing Centers in Selected Health Facilities in Bangladesh: Implementation Research
Source: JMIR Pediatr Parent. 2025 Jan 3;8:e65912. doi: 10.2196/65912 (PMC11748442; doi:10.2196/65912)
Supplement: Multimedia Appendix 3 [file pediatrics_v8i1e65912_app3.docx]

**Background information of the respondent:**

| Name: |  |
| --- | --- |
| ID number in quantitative survey: |  |

1. What encouraged you to start using the tele-mental health counselling?

- What motivated you?
- How would you describe your need which drove you to receive the tele-mental health counselling?
- How would you describe the role of the outpatient doctor, filed assistant, well-being corner in motivating you to receive the tele-mental health counselling?
- How would you describe the role of your family in motivating you to receive the tele-mental health counselling?
- Was there any barrier from the family or society in receiving this tele-mental health counselling? If yes, how would you describe those barriers?
- Did you discuss with anyone (family or relatives) once you were invited to the well-being corner for tele-mental health counselling? If yes, could you describe the discussion? With whom did you discuss? Have you been encouraged to receive the tele-mental health care?

1. How was your experience explaining your problem to the counsellors over videoconference tool?

- How would you describe your experience of sharing your words with the counsellor through videoconference tool?
- Did the counsellor listen to you carefully?
- How satisfied were you with sharing your problem to the counsellor?
- Did you feel safe while sharing your problem with the counsellor?
- How would you describe the attention of the counsellor towards you while you shared your problem?

1. What encouraged you to continue using the videoconference tool and receive the tele-mental health counselling?

- Would you receive the tele-mental health counselling again? If yes, what will motivate you to receive the service again?
- Probe: tele-mental health could solve my problem, I am satisfied with the overall quality of the service, the counsellors are amazing

1. What discouraged you from continue using videoconference tool and receive the tele-mental health counselling?

- Would you receive the tele-mental health counselling again? If no, what will demotivate you to receive the service again?
- Probe: tele-mental health could not solve my problem, I am not satisfied with the overall quality of the service, I do not like the counsellor.

1. How could the electronic therapy sessions delivered be improved?

- The audio, the appointments system, the access etc.

1. Would you choose to use the online therapy again in the future?
2. Is there anything else you’d like to say or share?
