# Supplementary material for: Enhancing Access to Mental Health Services for Antepartum and Postpartum Women Through Telemental Health Services at Wellbeing Centers in Selected Health Facilities in Bangladesh: Implementation Research
Source: JMIR Pediatr Parent. 2025 Jan 3;8:e65912. doi: 10.2196/65912 (PMC11748442; doi:10.2196/65912)
Supplement: Multimedia Appendix 4 [file pediatrics_v8i1e65912_app4.docx]

**Interview Guide**

1. Tell me about your role within the well-being corner.
   - How did you get involved in well-being corner, and what drew you to this current position?
   - What is in your opinion the purpose of the well-being corner?
   - Do you have other responsibilities besides this well-being corner?
2. Can you describe a typical working day?
   - What issues do you typically experience during your work in the well-being corner? Can you give some examples?
   - Are the majority of your activities planned or unplanned? (e.g., how often are you called for unscheduled events?)
3. Can you describe the relationship between yourself and the other colleagues and healthcare workers involved in the well-being corner?
   - How do you work together with them? What is the main method of communication within the well-being corner?
   - What issues do you typically encounter and how/who do you call for support?
   - Besides the hospital staff and counsellors, who else do you regularly work with? Can you describe to me how you work together?
4. Now I would like to talk about the videoconferencing sessions here in the clinic. Can you explain how a tele-mental health counselling session is organised?
5. In your opinion, what are the challenges you currently encounter when organising and delivering a tele-mental health videoconferencing counselling?
   - Conversely, what are, in your opinion, the advantages of videoconferencing compared to face-to-face interactions?
   - Do you believe that this videoconferencing technology could influence the therapeutic alliance? If so, can you elaborate?
   - Can you describe to me the process of what happens when there is a technical problem? How was it resolved?
6. Has an emergency ever occurred during a videoconferencing session before? Can you briefly describe to me what happened and how you dealt with it?
   - If not, how prepared do you think you are to deal with these potential emergencies?
7. I also know that you do/arrange both initial counselling and follow-up counselling by videoconferencing.
   - Is there a difference in the way you approach these two types of videoconferencing sessions? Can you explain?
   - Do you believe that it would be beneficial for all initial visits to be done in-person? Why or why not?
   - While doing the videoconferencing follow-ups, how do you know if a patient has improved *(not applicable for the facility manager)*?
8. According to the literature, missed videoconferencing counselling or follow-up counselling by patients are a significant issue.

- What do you do when a patient does not show up for their counselling?
- What do you think may be the reasons for patients to miss their videoconferencing appointment?

1. Tell me about the scheduling system for videoconferences. Is there anything that can be improved?
2. I have also learned that there are a few Northern patients who need to be hospitalised or referred to more advanced mental health support.
   - How challenging it is for you to understand which patients would need the follow-ups and which ones will not through video conferencing counselling?
   - How could you tell?
   - Compared to the face-to-face counselling, how would you describe the effective ness of the videoconferencing counselling.
3. Tell me about the documentation system that is currently being used.
   - Is there a specific software you are using?
     - Do you need to communicate with the other hospital staff and counsellors? If yes, why do you need to communicate. What are challenges do you face to communicate?
   - Is the documentation system universal, or is it different for the counsellors and hospital staff
   - What can be improved? What will be the advantages and disadvantages of implementing an electronic health record?
4. In your opinion, what are your recommendations to improve the well-being corner?
5. What do you think is the future of telehealth, mhealth for delivering tele-mental health services?
   - What are some future developments that you would like to see in the field of tele-mental health in the future?
     - Do you believe that the tele-mental health can adopt this in the future? Why or why not?
6. Is there anything else that you would like to talk about?

Thanks!
