# Supplementary figures and images for: Enhancing Access to Mental Health Services for Antepartum and Postpartum Women Through Telemental Health Services at Wellbeing Centers in Selected Health Facilities in Bangladesh: Implementation Research
Source: JMIR Pediatr Parent. 2025 Jan 3;8:e65912. doi: 10.2196/65912 (PMC11748442; doi:10.2196/65912)

**
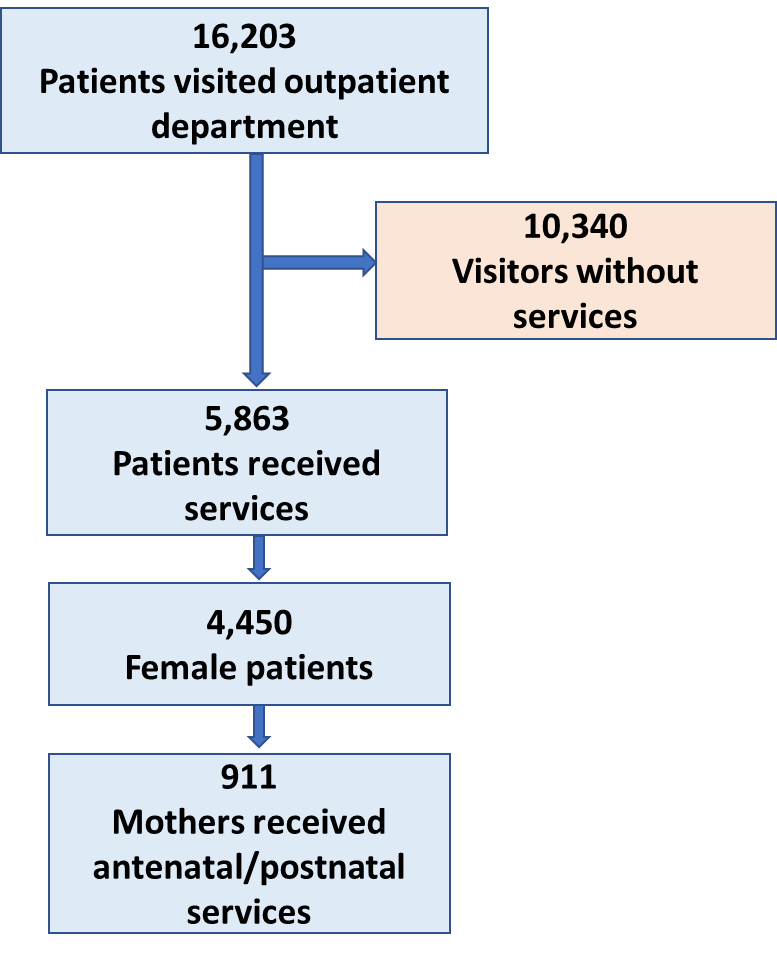
**

Supplement: Multimedia Appendix 5 [file pediatrics_v8i1e65912_app5.docx]

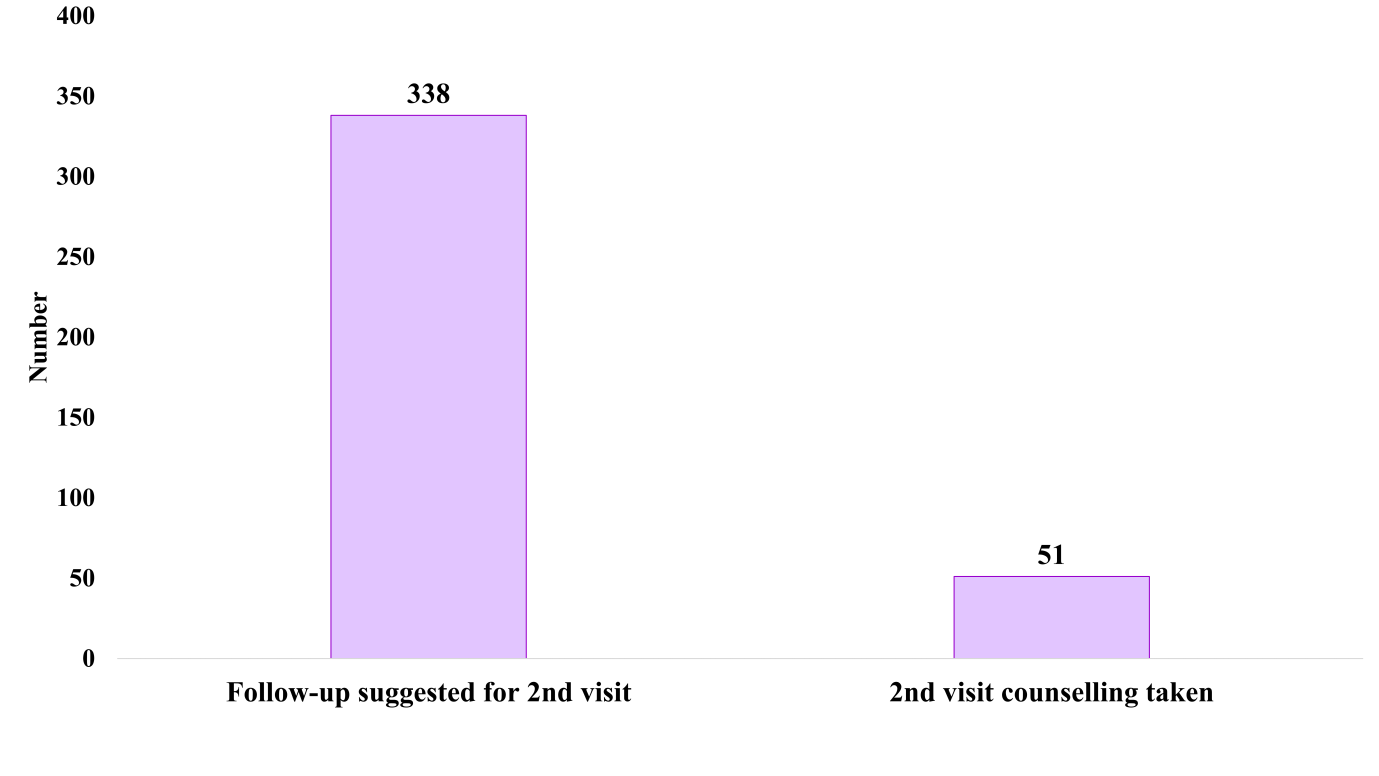

Supplement: Multimedia Appendix 6 [file pediatrics_v8i1e65912_app6.docx]
